# Prediction of the Topologically Nontrivial Phase in Three-Dimensional ABX Zintl Compounds

**DOI:** 10.1021/acsomega.4c08153

**Published:** 2025-01-02

**Authors:** Ina Marie R. Verzola, Rovi Angelo B. Villaos, Zhi-Quan Huang, Hsin Lin, Feng-Chuan Chuang

**Affiliations:** † Department of Physics, National Sun Yat-Sen University, Kaohsiung 80424, Taiwan; ‡ Institute of Physics, Academia Sinica, Taipei 115201, Taiwan; § Physics Division, National Center for Theoretical Sciences, Taipei 10617, Taiwan; ∥ Center for Theoretical and Computational Physics, National Sun Yat-Sen University, Kaohsiung 80424, Taiwan; ⊥ Department of Physics, National Tsing Hua University, Hsinchu 30013, Taiwan

## Abstract

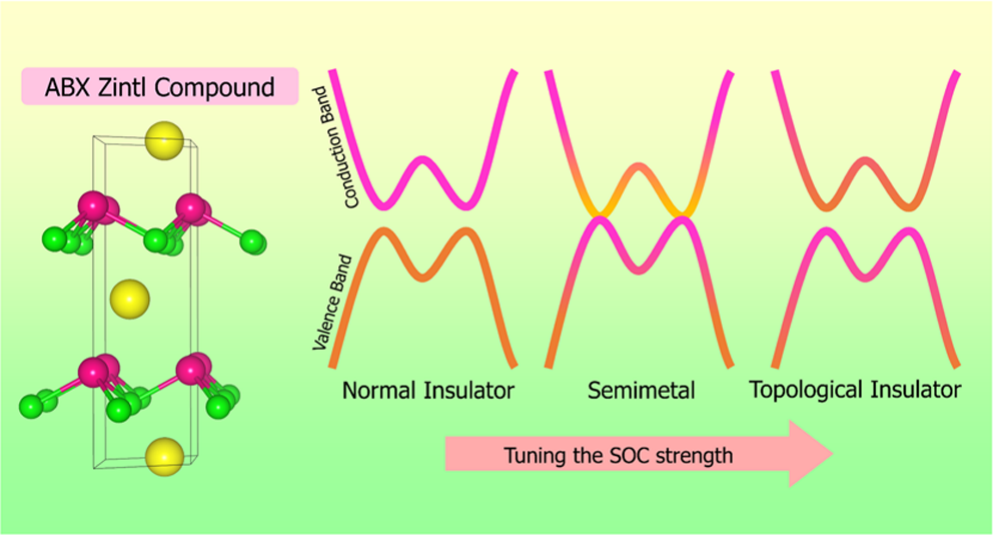

Zintl compounds have garnered research interest due to their diverse technological applications. Utilizing first-principles calculations, we performed a systematic study of ABX (A = Li, Na, K, Rb, or Cs; B = Si, Ge, Sn, or Pb; and X = P, As, Sb, or Bi) Zintl materials with the *P*6_3_*mc* KSnSb-type structure. Notably, six ABX Zintl compounds (RbSiBi, CsSiBi, LiGeBi, KGeBi, RbGeBi, and CsGeBi) were found to have topologically nontrivial phases, as demonstrated by the *Z*_2_ invariant computed using the hybrid functional HSE06. Among them, RbGeBi and CsGeBi were identified as topological insulators with nontrivial bandgaps of 28 and 116 meV, respectively. The topological phase transition arises as a result of spin–orbit coupling, as demonstrated in the representative material, CsGeBi. Additionally, the existence of gapless surface states further confirmed the topologically nontrivial phases of the six materials. Moreover, phonon spectra and formation energy calculations verified that all identified nontrivial materials under the hybrid functional are dynamically and structurally stable, except LiGeBi which exhibited imaginary phonon frequencies. Finally, the thermodynamic stability of the representative material CsGeBi was verified through elastic constants and ab initio molecular dynamics simulations. These results provide foundational insights that could drive further experimental research and synthesis, potentially enabling the application of ABX Zintl compounds in electronic technologies such as quantum computing or spintronics.

## 1 Introduction

A significant advancement in condensed matter physics is the discovery that spin–orbit coupling can generate an unconventional phase of matter taking the form of topological insulators (TIs). TIs are electronic materials characterized by a bulk bandgap, similar to conventional insulators, but they additionally feature protected conducting states on their surface or edge. According to the bulk-boundary correspondence, a *d*-dimensional TI has a (*d-1*)-dimensional topological boundary state.^1−4^ For example, three-dimensional (3D) TIs have two-dimensional (2D) Dirac surface states,^5,6^ and 2D TIs have one-dimensional (1D) helical boundary states.^7−9^ These surface states emerge as a result of the strong spin–orbit coupling (SOC) in these TI materials, which opens a bulk energy gap and forms conducting surface states. Numerous materials have demonstrated TI properties both theoretically and experimentally in recent years. Among these topological materials are, graphene,^10^ binary compounds in honeycomb and buckled honeycomb lattices,^11−13^ MXenes,^14−17^ transition metal dichalcogenides and halides,^18−21^ Janus materials,^22,23^ ternary transition metal chalcogenides,^24,25^ copper sulfide,^26^ silicene/SiC(0001),^27^ half-Heusler compounds,^28^ ilmenite oxides,^29^ MA_2_Z_4_ materials,^30−32^ and Zintl compounds.^33−35^

Among the aforementioned topological materials, complex-structured compounds such as Zintl materials are receiving research attention. Originally, Zintl structures have been explored for their thermoelectric features, with applications in power generation, renewable energy, and other technologies.^36^ Interestingly, certain thermoelectric materials with narrow bandgaps exhibit topological features because of their strong SOC effects.^37^ Recent studies have demonstrated that certain Zintl compounds indeed exhibit nontrivial topological phases.^38^ Various Zintl phase materials that exhibit nontrivial topological properties have been found in different structures, including a group of hexagonal layered compounds such as CaCuSb,^39^ LiZnSb,^40^ AM_2_X_2,_^41^ A_2_MX_2,_^42^ AIn_2_As_2,_^43,44^ and ABX that adopt the KSnSb-type structure.^45^ In particular, some ABX compounds with the KSnSb-type^45^ structure, such as KSnAs,^46^ NaSnP,^47^ and NaSnAs,^47^ have been experimentally synthesized. Recent theoretical studies have also shown that ABX Zintl compounds in the KSnSb structure have interesting nontrivial topological properties such as NaSnBi,^48^ KSnSb,^49^ KSnBi,^49^ RbSnBi,^50^ and CsSnBi^50^ which were identified to be strong topological insulators induced by SOC. In addition to the strong SOC effect, a topological phase transition in KSnSb and KSnBi can also be driven by external strain, such as compressive hydrostatic pressure.^49^ Furthermore, alloying the material KSnSb_1–*x*_Bi_*x*_ (*x* = 0.5) leads to the reopening of a topological gap, transforming the compound into a strong topological insulator.^49^ Looking forward, the ABX Zintl compounds present exciting opportunities for further research, particularly in areas such as tunable topological phase transitions. Although theoretical studies have explored the electronic and topological properties of certain bulk Zintl compounds, the broader family of ABX Zintl compounds remains underexplored in terms of their topological characteristics. A better understanding of the ABX Zintl family of compounds may identify new topological phases and broaden their potential applications in materials science.

Building on the extensive research into ABX Zintl compounds and their reported electronic and topological properties, an exhaustive materials search using first-principles calculations was conducted. Our study specifically examines the electronic and topological properties of ABX Zintl compounds where A is the alkali metals (Li, Na, K, Rb, or Cs), B represents metalloids (Si, Ge, Sn, or Pb), and X is the pnictogens (P, As, Sb, or Bi). Overall, 80 compounds were investigated. Interestingly, six compounds (RbSiBi, CsSiBi, LiGeBi, KGeBi, RbGeBi, and CsGeBi) were observed to have topologically nontrivial features as demonstrated by the *Z*_2_ number and surface states calculations using the hybrid functional HSE06. Additionally, most of these topologically nontrivial compounds were found to be dynamically and structurally stable. These findings highlight the potential of ABX Zintl materials exhibiting topologically nontrivial properties, which could significantly impact the field of Zintl topological materials and stimulate future theoretical and experimental investigations.

## 2 Computational Methods

First-principles calculations based on the framework of density functional theory^51^ were conducted through the Vienna Ab initio Simulation Package (VASP)^52,53^ with the projected augmented wave (PAW)^54,55^ in the Perdew–Burke–Ernzerhof (PBE) under the generalized gradient approximations (GGA)^56^ exchange-correlation functional. The plane-wave cutoff energy was set to 400 eV. The crystal structure was relaxed until the residual force on each atom was below 0.01 eV/Å. Moreover, the convergence criterion for self-consistent calculations was set to 10^–6^ eV. The first Brillouin zone was sampled using the Γ-centered Monkhorst–Pack^57^ grid of 18 × 18 × 6. The total ground-state energy and electronic band structure were computed using the PBE-GGA functional. Furthermore, the Heyd–Scuseria–Ernzerhof hybrid functional (HSE06)^58−60^ was implemented using a reduced Γ-centered Monkhorst–Pack grid of 9 × 9 × 3 to achieve a more accurate bandgap. Spin–orbit coupling (SOC) was taken into account in the calculations.

For the topological properties, WannierTools package^61^ was employed to calculate the *Z*_2_ topological invariants^62^ and surface states of the bulk ABX compounds using HSE06 via surface Green’s function within the framework of the tight-binding model by constructing maximally localized Wannier functions (MLWF) obtained from the Wannier90^63^ program. The *Z*_2_ topological invariant is denoted as (*v*_0_; *v*_1_*v*_2_*v*_3_), where *v*_0_, *v*_1_, *v*_2_, and *v*_3_ are the four distinct *Z*_2_ invariants.^62,64^ The value of *v*_0_ is indicated as *v*_0_ = [*Z*_2_(*k*_*i*_ = 0) + *Z*_2_(*k*_*i*_ = 0.5)]*mod*2, while *v*_*i*_ = *Z*_2_(*k*_*i*_ = 0.5), for *i* = 1, 2, 3. A schematic illustration of these invariants is shown in Figure S1. Topological materials are categorized as strong topological insulators (TI), weak TI, and normal insulators according to the values of the four invariants. A material is classified as a strong TI if *v*_0_ = 1. If *v*_0_ = 0, and at least one of *v*_1_, *v*_2_, and *v*_3_ is nonzero, then the material is categorized as a weak TI. If all four invariants are zero, the material is considered to have a trivial topology.

To establish dynamic stability, phonon dispersion spectra were calculated using the Phonopy package,^65^ with a large supercell size of 3 × 3 × 2 for the force calculations. Elastic constants and mechanical properties were obtained from the second-order derivatives of the total energy versus applied strain using VASPKIT.^66^ Thermal stability was assessed through ab initio molecular dynamics (AIMD) simulations within the canonical ensemble (NVT) framework, utilizing the Nosé–Hoover thermostat.^67−69^ The AIMD simulations were conducted with a 2 × 2 × 2 supercell for a duration of 6 ps, using a time step of 1 fs at a temperature of 300 K. The *k*-point sampling was limited to the Γ-point.

## 3 Results and Discussion

**Figure 1:**
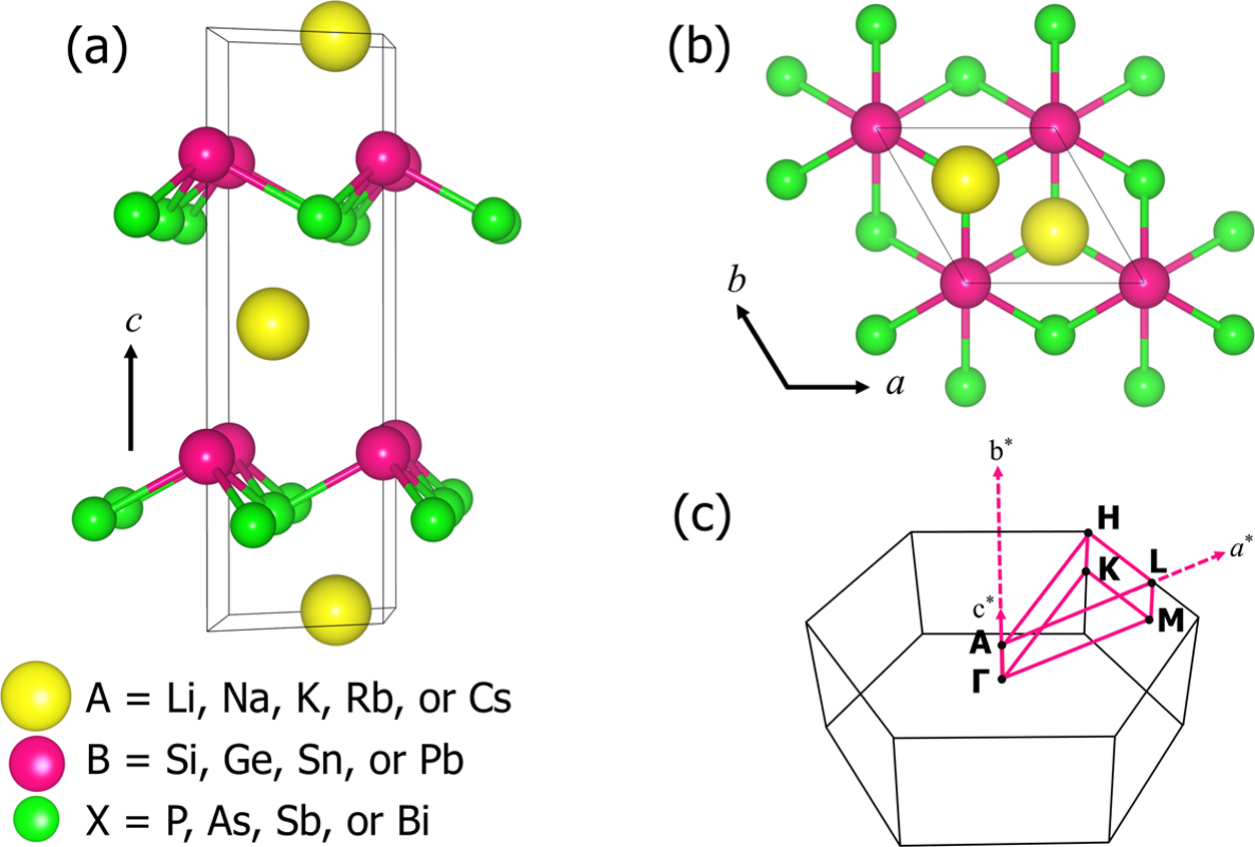
(a) Perspective and (b) top views of the crystal structure of ABX Zintl compounds (A = Li, Na, K, Rb, or Cs; B = Si, Ge, Sn, or Pb; X = P, As, Sb, or Bi). The yellow, pink, and green spheres represent the alkali metal (A), metalloid (B), and pnictide (X), respectively. (c) The first Brillouin zone (BZ) of the ABX Zintl structure, along the high-symmetry points and the reciprocal lattice vectors a*, b*, and c*.

The structure of the ABX Zintl compounds belongs to the hexagonal space group of *P*6_3_*mc* (number 186) and features a noncentrosymmetric layered arrangement along the *z*-direction, as illustrated in Figure 1. This structure is based on experimentally synthesized KSnAs and KSnSb compounds.^46^ The A, B, and X elements are alkali metals, metalloids, and pnictides, respectively. In a unit cell, there are two alternately stacked A–B–X layers, where B and X atoms form strong covalent bonds, while A and B atoms form ionic bonds. Figure 1a,b depict the atomic structure from different perspectives, while Figure 1c illustrates its 3D first Brillouin zone (BZ). The relaxed lattice parameters of ABX Zintl compounds are summarized in Tables S1–S4, demonstrating close agreement with experimental values.

Using the aforementioned ABX Zintl structure, the electronic and topological properties of 80 ABX Zintl compounds were studied through an exhaustive search approach.^30^ As an initial search protocol, the electronic structures of the materials were calculated using PBE-GGA functional, as shown in Figures S2–S17. The materials were classified as either metallic or semiconducting after incorporating SOC. For materials exhibiting an energy gap between the valence band maximum (VBM) and conduction band minimum (CBM), the *Z*_2_ number was calculated using PBE-GGA.

The *Z*_2_ numbers for the 80 ABX Zintl compounds calculated under PBE-GGA are provided in Table S5. In this initial screening, 13 out of the 80 compounds considered exhibit nontrivial topological phases. However, since the nontrivial topological properties of four of these materials (NaSnBi,^48^ KSnBi,^49^ RbSnBi,^50^ and CsSnBi^50^) are already well-studied, we focused on the remaining nine materials listed in Table 1. To improve accuracy and avoid underestimating bandgaps, hybrid functional (HSE06) calculations were performed for these selected compounds.^70,71^ Out of the nine topologically nontrivial compounds, six retained their topological characteristics namely RbSiBi, CsSiBi, LiGeBi, KGeBi, RbGeBi, and CsGeBi. Interestingly, RbGeBi and CsGeBi were identified as strong topological insulators with positive system bandgaps of 28 and 116 meV, respectively, while the remaining four compounds are strong topological materials with negative system bandgaps. Table 1 summarizes the calculated bandgaps of the selected ABX Zintl compounds.

**Table 1:** Topological Invariants, System Bandgaps, and Bandgaps at the Γ Point for the Selected Nine Bulk ABX Compounds, Calculated Using HSE06 with SOC^a^

ABX	*Z*_2_ (PBE-GGA)	*Z*_2_ (HSE06)	Material Classification (HSE06)	System Bandgap (meV)	Bandgap at Γ (meV)
KSiBi	(1;111)	(0;000)	normal insulator	121	171
RbSiBi	(1;111)	(1;000)	strong topological material	–32	18
CsSiBi	(1;111)	(1;000)	strong topological material	–12	277
LiGeBi	(1;111)	(1;000)	strong topological material	–920	920
NaGeBi	(1;111)	(0;000)	normal semimetal	–243	318
KGeBi	(1;111)	(1;000)	strong topological material	–12	133
RbGeBi	(1;111)	(1;000)	strong topological insulator	28	244
CsGeBi	(1;111)	(1;000)	strong topological insulator	116	544
LiSnBi	(1;111)	(0;000)	normal semimetal	–542	1616

a The *Z*_2_ number is represented as (*v*_0_; *v*_1_*v*_2_*v*_3_). A negative system bandgap indicates a semimetal, where the top of the valence band is higher in energy than the bottom of the conduction band. A positive system bandgap indicates the material is an insulator.

**Figure 2:**
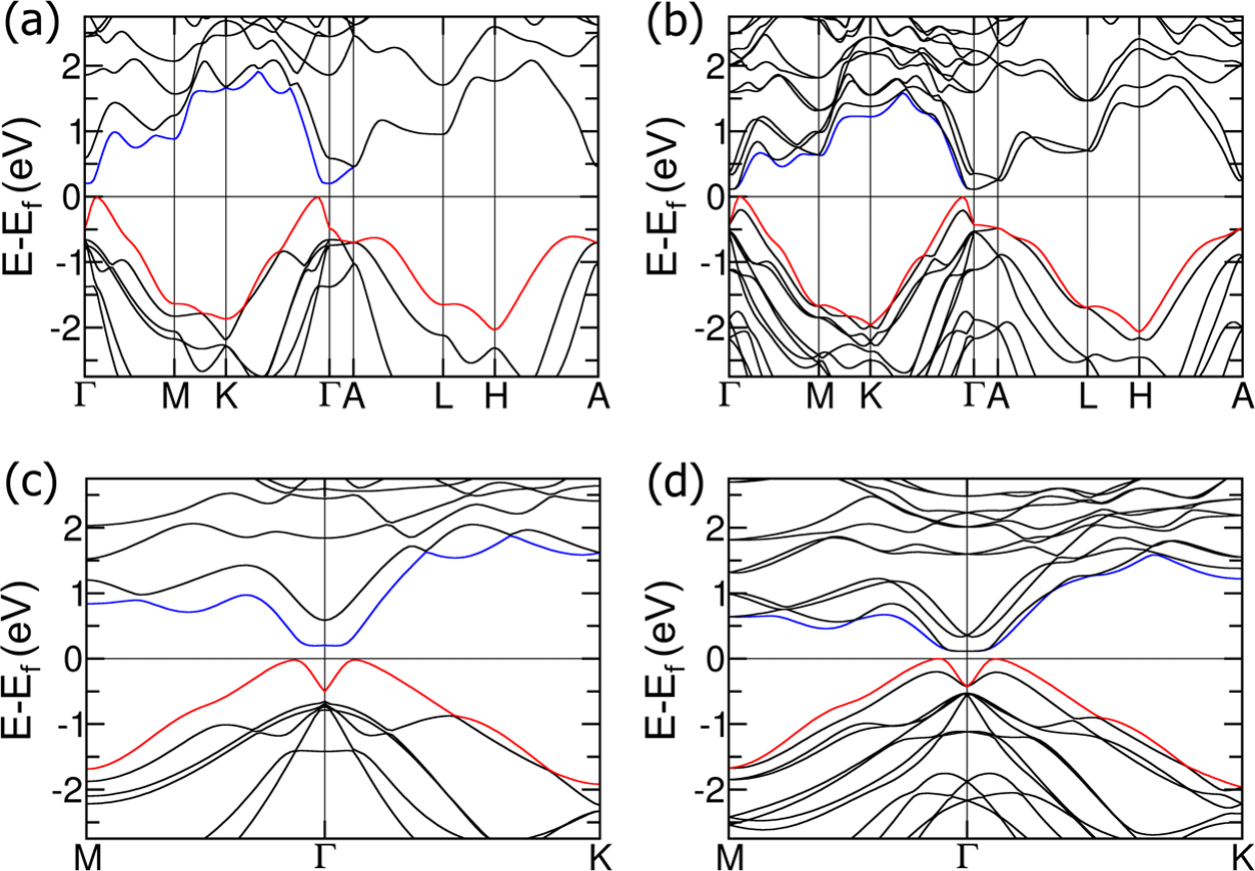
Electronic band structures of CsGeBi under HSE06 (a) without SOC, (b) with SOC, and along the *M*–Γ–*K* path (c) without SOC and (d) with SOC. The red and blue bands represent the valence band maximum (VBM) and conduction band minimum (CBM), respectively.

We further investigated the electronic and topological features of the nine topologically nontrivial ABX Zintl compounds using HSE06 to accurately obtain the electronic bandgaps and topologically nontrivial features, with a focus on topological insulators. Figures 2 and S18–S25 present the band structures. CsGeBi was selected as the representative material, as it has the largest positive bandgap among the topologically nontrivial materials, as summarized in Table 1. The PBE-GGA bandgaps of the other studied materials are summarized in Tables S6–S9. Figure 2a,b display the band structures of CsGeBi without and with spin–orbit coupling (SOC), respectively, where the VBM is colored in red and the CBM is colored in blue. Without SOC, CsGeBi is a narrow-gap semiconductor featuring an indirect system bandgap of 171 meV and a bandgap at Γ of 707 meV. Upon including SOC, the indirect system bandgap reduces to 116 meV, and the bandgap at Γ narrows to 544 meV. The calculated bandgap of CsGeBi is comparable to those of well-known room-temperature topological insulators such as Bi_2_Se_3_^72^ (∼220 meV) and Sb_2_Te_3_^73^ (∼190 meV). Therefore, CsGeBi could be a promising candidate for applications requiring thermal stability at room temperature while maintaining nontrivial topological properties.

**Figure 3:**
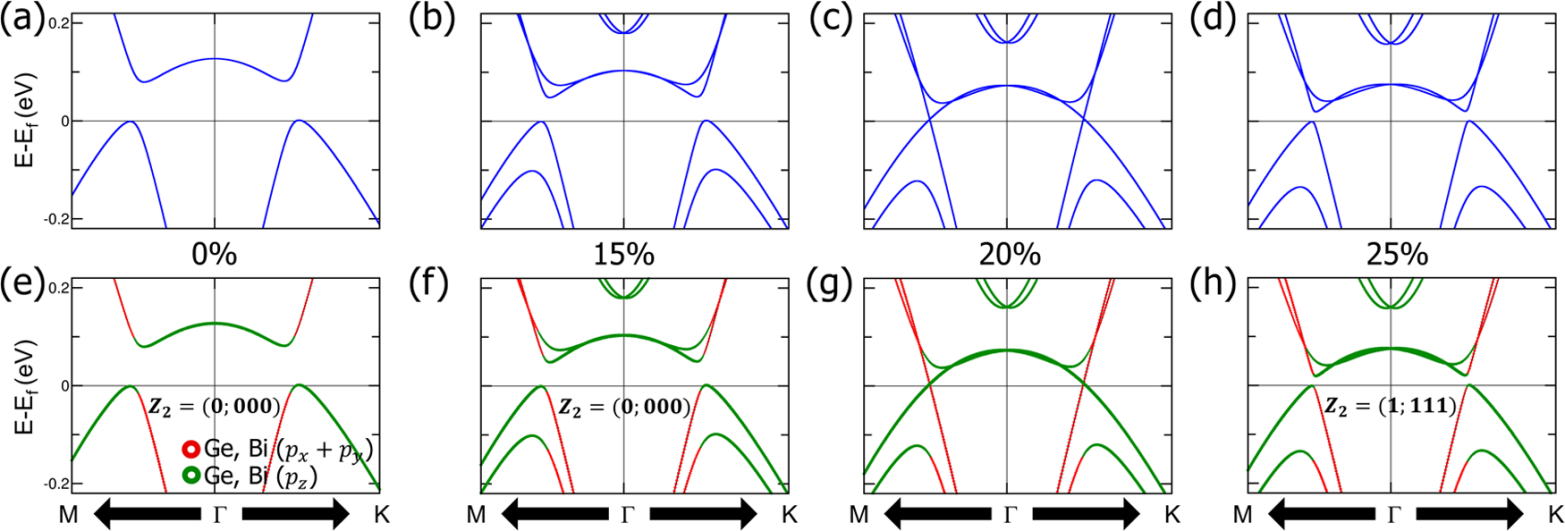
Effect of varying the spin–orbit coupling (SOC) strength on the band structures and topological *Z*_2_ number of CsGeBi under PBE-GGA. The band structures of CsGeBi with (a) 0%, (b) 15%, (c) 20%, and (d) 25% of the actual atomic SOC. The corresponding orbital characteristics for each case are presented in (e–h).

Topological phase transition is an important concept in topological band theory, which occurs when an originally open gap is closed and then reopened, typically induced by SOC. To elucidate the mechanism behind the topological phase transition in CsGeBi, we conduct calculations by varying the SOC strength from 0 to the actual atomic value, following the procedures used in NaSnBi.^48^ We focus on the gap at Γ. The weight of the p_*x*_ + p_*y*_ and p_*z*_ orbitals of Ge and Bi atoms illustrates the interchange of their band characteristics, as presented in Figure 3e–h. Figure 3a,e display the band structures without SOC. Interestingly, p_*x*_ + p_*y*_ and p_*z*_ band inversion occurs in CsGeBi even without SOC due to crystal-field splitting, analogous to that observed in previously published ASnBi (A = Na, K, Rb, or Cs)^48−50^ Zintl compounds, which are strong topological insulators. When SOC is included, there is significant splitting of both the VBM and CBM around Γ because of the large SOC of Bi, as shown in Figure 3b–d,f,g. Additionally, the band inversion is observed in spin-split bands. The band structures with SOC strengths adjusted from 15 to 25% are shown in Figure 3f,g, while a more detailed evolution of the electronic band structures from 0 to 100% SOC strength is depicted in Figure S26. At 15% SOC, as observed in Figure 3f, gaps appear along the *M*–Γ and Γ–*K* directions. When the SOC strength is increased to 20%, a band touch occurs along the *M*–Γ and Γ–*K* paths, as presented in Figure 3g. Further increasing the SOC strength reopens the gap, as illustrated in Figure 3h. Hence, the gaps along the *M*–Γ and Γ–*K* paths decrease with increasing SOC until they close completely at a threshold SOC strength, after which the gaps reopen as the SOC strength continues to increase. The CsGeBi compound remains in a topologically trivial phase when the SOC strength is less than 20% and transitions to a topologically nontrivial phase when the SOC strength exceeds 20%. These observations demonstrate that the CsGeBi compound can transition into a topological phase as the SOC strength is increased.

To confirm the topologically nontrivial phase, the *Z*_2_ number was calculated using the WannierTools package.^61^ The *Z*_2_ invariant calculated for CsGeBi is (1;000), indicating that it is indeed a strong topological insulator. Additionally, RbSiBi, CsSiBi, LiGeBi, KGeBi, and RbGeBi also have *Z*_2_ invariants of (1;000), as summarized in Table 1, indicating that these five materials are strong topological materials. In contrast, the *Z*_2_ invariants for KSiBi, NaGeBi, and LiSnBi are (0;000), which classifies them as trivial materials. These materials are categorized as either normal semimetals if they possess a negative system bandgap or normal insulators if they have a positive system bandgap.

**Figure 4:**
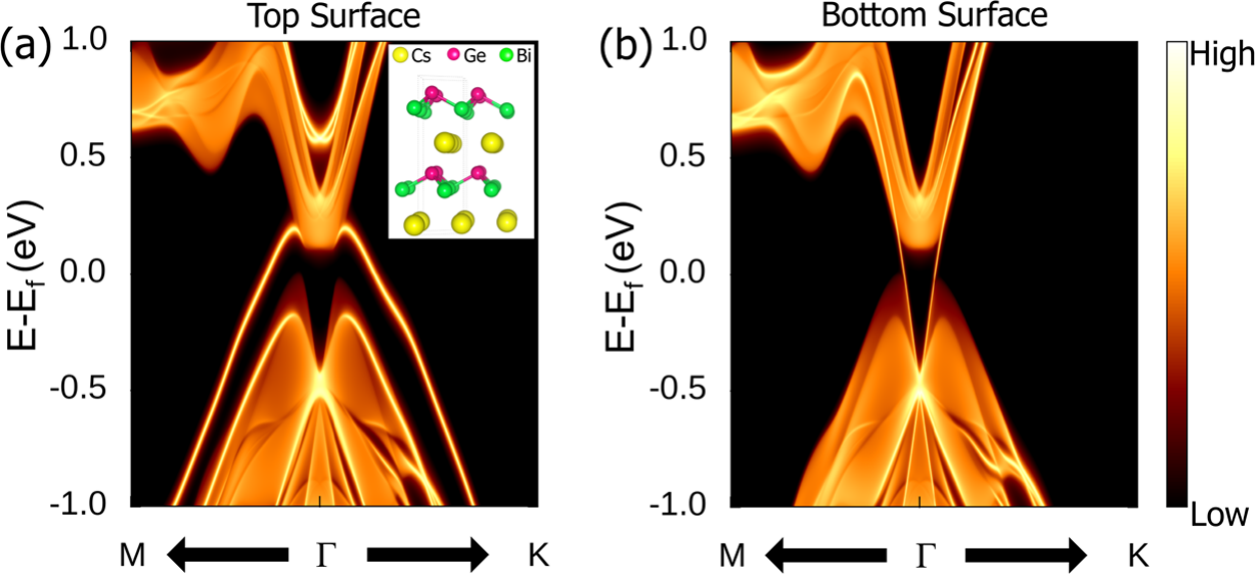
Surface electronic spectrum of CsGeBi under HSE06 at (a) the top surface (Ge-terminated) and (b) the bottom surface (Cs-terminated) along the (001) surface. The inset in (a) illustrates the structure termination, with the top surface being Ge-terminated and the bottom surface being Cs-terminated.

To further investigate the nontrivial topological properties of the ABX Zintl compounds, Green’s function-derived surface states on the (001) surface were calculated via the WannierTools.^61^Figure S27 presents the Wannier band structure along with the orbital projections utilized for the Wannier fitting. A key feature of nontrivial topology is the existence of linear dispersion within the bulk gap, indicating gapless surface states. To examine the surface states in CsGeBi, a semi-infinite slab structure with two types of surface termination was considered. In particular, the top surface is Ge-terminated, while the bottom surface is Cs-terminated, as shown in the inset of Figure 4a. Experimentally, surface terminations can be achieved through techniques such as cleaving, commonly used for layered materials. Cleaving is typically performed in an ultrahigh vacuum to expose clean surfaces along cleavage planes.^74^ Additionally, scanning tunneling microscopy (STM) or angle-resolved photoemission spectroscopy (ARPES) can be employed to verify the nature of the surface terminations after cleaving.^75^ Interestingly, as shown in Figure 4a, a non-Dirac-like dispersion appears at the top surface (Ge-terminated). In contrast, Figure 4b displays a Dirac-like spectrum at the bottom surface (Cs-terminated), illustrating the gapless surface states that span the bulk energy gap connecting the conduction band and valence band presented in Figure 2d. Thus, the computational simulation of the surface states of CsGeBi indicates the existence of nontrivial surface states at the Fermi level, confirming it as a strong topological insulator. Moreover, computational simulations of surface states in RbSiBi, CsSiBi, LiGeBi, KGeBi, and RbGeBi also indicate the existence of nontrivial surface states at the Fermi level, as shown in Figures S28–S32.

**Figure 5:**
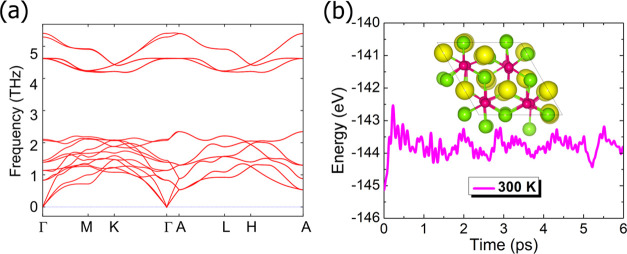
(a) Phonon dispersion curves of CsGeBi. (b) Total energy fluctuations with time during the ab initio molecular dynamics (AIMD) simulation of CsGeBi at 300 K. The structure observed during the 6 ps simulation is shown in the inset.

Finally, since this study primarily focuses on the topological features, the structural stability of the six topologically nontrivial ABX Zintl compounds was explored. The dynamic stabilities of the RbSiBi, CsSiBi, LiGeBi, KGeBi, RbGeBi, and CsGeBi compounds were examined through phonon frequencies calculated at the high-symmetry points of the BZ, as shown in Figures 5a and S33. All phonon curves were found to be positive throughout the BZ, suggesting that the phonon branches are free from imaginary frequencies, except for LiGeBi. Hence, the phonon dispersion spectra confirm the dynamic stability of the topologically nontrivial ABX Zintl systems (RbSiBi, CsSiBi, KGeBi, RbGeBi, and CsGeBi) and their potential to exist as stable materials. Structural stability was further assessed by calculating the formation energy of these topologically nontrivial ABX Zintl systems. The negative formation energy values presented in Table S10 indicate the stability of the six nontrivial compounds.

Moreover, to assess the mechanical stability of the representative material, CsGeBi, its elastic constants were calculated and are summarized in Table S11. The mechanical stability criteria for the hexagonal system are as follows:^76^*C*_11_ > |*C*_12_|, 2*C*_13_^2^ < *C*_33_(*C*_11_ + *C*_12_), *C*_44_ > 0, *C*_66_ > 0. Table S11 indicates that the elastic constants satisfy these criteria, demonstrating that the CsGeBi Zintl compound is stable at the mechanical level. Lastly, to verify the thermal stability of CsGeBi, ab initio molecular dynamics (AIMD) simulations were conducted at 300 K for 6 ps. Figure 5b displays small fluctuations in the total energy, with atoms vibrating around their equilibrium positions without significant structural changes. The inset structure indicates that the bonds between atoms remain intact, confirming that CsGeBi is thermally stable at 300 K. Thus, the structure of CsGeBi exhibits thermodynamic stability. Since these six compounds have not yet been experimentally reported, these calculations could provide valuable insights for experimentalists considering the synthesis of these topologically nontrivial materials in the laboratory. The two-step process used in the preparation of KSnAs and KSnSb,^46^ which starts with a binary compound (KSn) and reacting it with a third element (As or Sb), can serve as a template for the synthesis of other ABX Zintl compounds. By selecting suitable starting materials for the A, B, and X components, researchers could adopt similar methodologies to create a wide range of ABX materials.

## 4 Conclusions

Our investigation focused on the structural stability, electronic properties, and topological characteristics of bulk ABX Zintl compounds (A is the alkali metals Li, Na, K, Rb, or Cs; B represents the metalloids Si, Ge, Sn, or Pb; and X is pnictogens P, As, Sb, or Bi). Among the 80 materials studied, six ABX Zintl compounds (RbSiBi, CsSiBi, LiGeBi, KGeBi, RbGeBi, and CsGeBi) exhibit strong topologically nontrivial properties under the hybrid functional HSE06. Our findings indicate that the topological phase transition in the representative compound, CsGeBi, is driven by strong spin–orbit coupling. Additionally, the calculated surface states of CsGeBi manifest gapless features, further confirming its strong nontrivial topological phase. The stability of these topologically nontrivial ABX Zintl compounds is supported by phonon dispersion, formation energy, elastic constants, and ab initio molecular dynamics calculations, except LiGeBi which displayed imaginary phonon frequencies. These results suggest that the new ABX Zintl family possesses topologically nontrivial properties that have substantial potential to stimulate further theoretical and experimental research in materials science and condensed matter physics.

## Data Availability

The data that support the findings of this study are available within the article and its Supporting Information.

## Supporting Information Available

Optimized lattice parameters; *Z*_2_ topological invariant number; calculated band gaps; band structures using PBE-GGA and HSE06; calculated edge states; phonon dispersion spectra; formation energy; mechanical properties (PDF)
