# Supplementary material for: Prediction of the Topologically Nontrivial Phase in Three-Dimensional ABX Zintl Compounds
Source: ACS Omega. 2025 Jan 2;10(1):964–71. doi: 10.1021/acsomega.4c08153 (PMC11740378; doi:10.1021/acsomega.4c08153)
Supplement: Supplementary file 1 — ao4c08153_si_001.pdf [file ao4c08153_si_001.pdf]

Supporting Information for

Prediction of the topologically nontrivial phase in three-dimensional ABX Zintl compounds

Ina Marie R. Verzola[1], Rovi Angelo B. Villaos[1, 2], Zhi-Quan Huang[1], Hsin Lin[2], and Feng-Chuan Chuang[1, 3, 4, 5, *]

[1]*Department of Physics, National Sun Yat-sen University, Kaohsiung, 80424 Taiwan*

[2]*Institute of Physics, Academia Sinica, Taipei, 115201, Taiwan*

[3]*Physics Division, National Center for Theoretical Sciences, Taipei, 10617 Taiwan*

[4]*Center for Theoretical and Computational Physics, National Sun Yat-sen University, Kaohsiung, 80424 Taiwan*

[5]*Department of Physics, National Tsing Hua University, Hsinchu, 30013 Taiwan*

*Corresponding Author: fchuang@mail.nsysu.edu.tw

Postal Address: 70 Lienhai Rd., Kaohsiung 80424, Taiwan.

Telephone: +886-7-5253733

1. Structural Parameters and Stability

1.1. Optimized Lattice Parameters of ABX Zintl Compounds

Table S1. The relaxed lattice parameters of the ABP compounds (A = Li, Na, K, Rb, or Cs; B = Si, Ge, Sn, or Pb) in units of Å. The values enclosed in parentheses are experimental lattice parameters.

**ABX**	**Lattice Parameter (Å)**		**ABX**	**Lattice Parameter (Å)**	
	**a = b**	**c**		**a = b**	**c**
**LiSiP**	3.469	10.308	**LiSnP**	3.877	10.388
**NaSiP**	3.541	11.506	**NaSnP**	3.905<br>(3.878)[47]	11.612<br>(11.666)[47]
**KSiP**	3.648	12.558	**KSnP**	4.029	12.833
**RbSiP**	3.702	13.077	**RbSnP**	4.079	13.366
**CsSiP**	3.762	13.549	**CsSnP**	4.144	13.874
**LiGeP**	3.605	10.103	**LiPbP**	4.194	8.894
**NaGeP**	3.663	11.376	**NaPbP**	4.105	11.404
**KGeP**	3.778	12.445	**KPbP**	4.193	12.618
**RbGeP**	3.833	12.957	**RbPbP**	4.244	13.199
**CsGeP**	3.910	13.427	**CsPbP**	4.310	13.774

Table S2. The relaxed lattice parameters of the ABAs compounds (A = Li, Na, K, Rb, or Cs; B = Si, Ge, Sn, or Pb) in units of Å. The values enclosed in parentheses are experimental lattice parameters.

ABX	Lattice Parameter (Å)		ABX	Lattice Parameter (Å)	
	a = b	c		a = b	c
**LiSiAs**	3.635	10.410	**LiSnAs**	4.129	9.421
**NaSiAs**	3.699	11.654	**NaSnAs**	4.050 (4.000)[47]	11.627 (11.728)[47]
**KSiAs**	3.814	12.715	**KSnAs**	4.179 (4.103)[46]	12.927 (12.845)[46]
**RbSiAs**	3.860	13.211	**RbSnAs**	4.233	13.393
**CsSiAs**	3.928	13.693	**CsSnAs**	4.305	13.806
**LiGeAs**	3.802	9.799	**LiPbAs**	4.402	8.421
**NaGeAs**	3.818	11.480	**NaPbAs**	4.285	11.093
**KGeAs**	3.934	12.554	**KPbAs**	4.350	12.668
**RbGeAs**	3.989	13.060	**RbPbAs**	4.398	13.164
**CsGeAs**	4.070	13.525	**CsPbAs**	4.439	13.687

Table S3. The relaxed lattice parameters of the ABSb compounds (A = Li, Na, K, Rb, or Cs; B = Si, Ge, Sn, or Pb) in units of Å. The values enclosed in parentheses are experimental lattice parameters.

ABX	Lattice Parameter (Å)		ABX	Lattice Parameter (Å)	
	a = b	c		a = b	c
**LiSiSb**	3.936	10.572	**LiSnSb**	4.518	8.900
**NaSiSb**	3.961	11.974	**NaSnSb**	4.353	11.676
**KSiSb**	4.083	13.185	**KSnSb**	4.445<br>(4.359)[46]	13.240<br>(13.150)[46]
**RbSiSb**	4.130	13.685	**RbSnSb**	4.495	13.604
**CsSiSb**	4.188	14.195	**CsSnSb**	4.546	14.235
**LiGeSb**	4.065	10.110	**LiPbSb**	4.800	7.994
**NaGeSb**	4.080	11.865	**NaPbSb**	4.609	10.777
**KGeSb**	4.182	13.024	**KPbSb**	4.620	12.854
**RbGeSb**	4.243	13.526	**RbPbSb**	4.635	13.353
**CsGeSb**	4.311	13.934	**CsPbSb**	4.692	13.867

Table S4. The relaxed lattice parameters of the ABBi compounds (A = Li, Na, K, Rb, or Cs; B = Si, Ge, Sn, or Pb) in units of Å.

ABX	Lattice Parameter (Å)		ABX	Lattice Parameter (Å)	
	a = b	c		a = b	c
**LiSiBi**	4.064	10.621	**LiSnBi**	4.665	8.709
**NaSiBi**	4.091	12.008	**NaSnBi**	4.498	11.556
**KSiBi**	4.209	13.303	**KSnBi**	4.559	13.276
**RbSiBi**	4.260	13.680	**RbSnBi**	4.586	13.488
**CsSiBi**	4.322	14.032	**CsSnBi**	4.642	14.054
**LiGeBi**	4.192	10.094	**LiPbBi**	4.940	7.905
**NaGeBi**	4.213	11.838	**NaPbBi**	4.786	10.165
**KGeBi**	4.290	13.086	**KPbBi**	4.726	12.868
**RbGeBi**	4.341	13.610	**RbPbBi**	4.676	13.309
**CsGeBi**	4.399	14.115	**CsPbBi**	4.710	13.603

2. Topological Properties of ABX Zintl Compounds

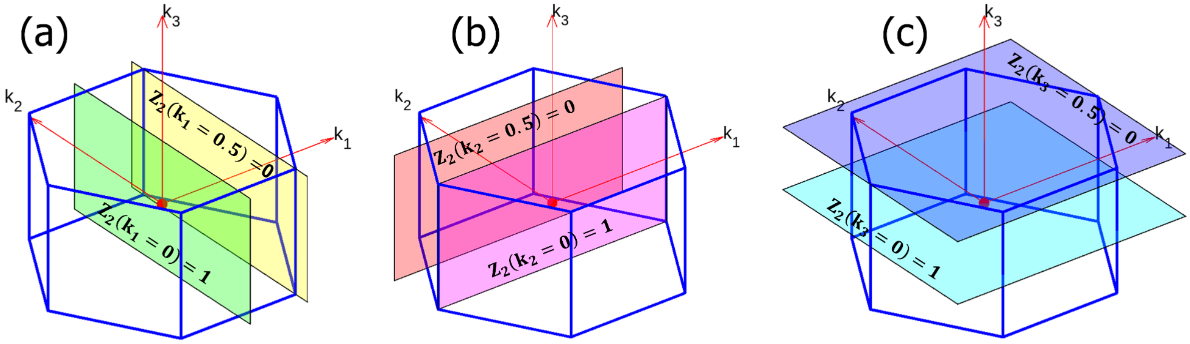

Figure S1. Schematic diagram of Z_2 topological invariants. The colored two-dimensional planes in the three-dimensional first Brillouin zone represent the time-reversal invariant planes of CsGeBi. Each plane is assigned a Z_2 topological number along (a) $k_1 = 0$ and $k_1 = 0.5$; (b) $k_2 = 0$ and $k_2 = 0.5$; and (c) $k_3 = 0$ and $k_3 = 0.5$.

Table S5. List of topological properties of ABX Zintl compounds under PBE-GGA with SOC. The Z_2 number Z_2 = (0;000) indicates the trivial phase while Z_2 = (1;111) denotes the strong nontrivial topological phase of the materials. On the other hand, metallic systems are denoted as M since the Z_2 invariant does not apply to metals.

ABX	Z_2 Invariant (PBE-GGA)	ABX	Z_2 Invariant (PBE-GGA)	ABX	Z_2 Invariant (PBE-GGA)	ABX	Z_2 Invariant (PBE-GGA)
**LiSiP**	(0;000)	**LiSiAs**	(0;000)	**LiSiSb**	(0;000)	**LiSiBi**	(0;000)
**NaSiP**	(0;000)	**NaSiAs**	(0;000)	**NaSiSb**	(0;000)	**NaSiBi**	(0;000)
**KSiP**	(0;000)	**KSiAs**	(0;000)	**KSiSb**	(0;000)	**KSiBi**	(1;111)
**RbSiP**	(0;000)	**RbSiAs**	(0;000)	**RbSiSb**	(0;000)	**RbSiBi**	(1;111)
**CsSiP**	(0;000)	**CsSiAs**	(0;000)	**CsSiSb**	(0;000)	**CsSiBi**	(1;111)
**LiGeP**	(0;000)	**LiGeAs**	(0;000)	**LiGeSb**	(0;000)	**LiGeBi**	(1;111)
**NaGeP**	(0;000)	**NaGeAs**	(0;000)	**NaGeSb**	(0;000)	**NaGeBi**	(1;111)
**KGeP**	(0;000)	**KGeAs**	(0;000)	**KGeSb**	(0;000)	**KGeBi**	(1;111)
**RbGeP**	(0;000)	**RbGeAs**	(0;000)	**RbGeSb**	(0;000)	**RbGeBi**	(1;111)
**CsGeP**	(0;000)	**CsGeAs**	(0;000)	**CsGeSb**	(0;000)	**CsGeBi**	(1;111)
**LiSnP**	(0;000)	**LiSnAs**	(0;000)	**LiSnSb**	(0;000)	**LiSnBi**	(1;111)
**NaSnP**	(0;000)	**NaSnAs**	(0;000)	**NaSnSb**	(0;000)	**NaSnBi**	(1;111)
**KSnP**	(0;000)	**KSnAs**	(0;000)	**KSnSb**	(0;000)	**KSnBi**	(1;111)
**RbSnP**	(0;000)	**RbSnAs**	(0;000)	**RbSnSb**	(0;000)	**RbSnBi**	(1;111)
**CsSnP**	(0;000)	**CsSnAs**	(0;000)	**CsSnSb**	(0;000)	**CsSnBi**	(1;111)
**LiPbP**	(0;000)	**LiPbAs**	(0;000)	**LiPbSb**	(0;000)	**LiPbBi**	M
**NaPbP**	(0;000)	**NaPbAs**	(0;000)	**NaPbSb**	(0;000)	**NaPbBi**	(0;000)
**KPbP**	(0;000)	**KPbAs**	(0;000)	**KPbSb**	(0;000)	**KPbBi**	M
**RbPbP**	(0;000)	**RbPbAs**	(0;000)	**RbPbSb**	(0;000)	**RbPbBi**	M
**CsPbP**	(0;000)	**CsPbAs**	(0;000)	**CsPbSb**	(0;000)	**CsPbBi**	M

3. Electronic Properties of ABX Zintl Compounds

3.1. System Bandgaps

Table S6. The system bandgaps of the ABP compounds (A = Li, Na, K, Rb, or Cs; B = Si, Ge, Sn, or Pb) under PBE-GGA without and with SOC. The positive system bandgap denotes a semiconducting nature while the negative one denotes a semimetallic nature.

ABX	System Bandgaps (eV)		ABX	System Bandgaps (eV)	
	without SOC	with SOC		without SOC	with SOC
**LiSiP**	0.591	0.588	**LiSnP**	0.044	0.023
**NaSiP**	0.772	0.769	**NaSnP**	0.562	0.536
**KSiP**	0.769	0.874	**KSnP**	0.798	0.784
**RbSiP**	0.613	0.611	**RbSnP**	0.606	0.587
**CsSiP**	0.000	-0.010	**CsSnP**	0.530	0.498
**LiGeP**	0.246	0.232	**LiPbP**	0.014	-0.108
**NaGeP**	0.556	0.545	**NaPbP**	0.296	0.198
**KGeP**	0.764	0.755	**KPbP**	0.526	0.425
**RbGeP**	0.487	0.474	**RbPbP**	0.535	0.423
**CsGeP**	0.400	0.373	**CsPbP**	0.616	0.468

Table S7. The system bandgaps of the ABAs compounds (A = Li, Na, K, Rb, or Cs; B = Si, Ge, Sn, or Pb) under PBE-GGA without and with SOC. The positive system bandgap denotes a semiconducting nature while the negative one denotes a semimetallic nature.

**ABX**	**System Bandgaps (eV)**		**ABX**	**System Bandgaps (eV)**	
	**without SOC**	**with SOC**		**without SOC**	**with SOC**
**LiSiAs**	0.191	0.163	**LiSnAs**	-0.764	-0.826
**NaSiAs**	0.504	0.478	**NaSnAs**	0.193	0.136
**KSiAs**	0.682	0.651	**KSnAs**	0.332	0.277
**RbSiAs**	0.413	0.386	**RbSnAs**	0.311	0.248
**CsSiAs**	0.195	0.148	**CsSnAs**	0.412	0.316
**LiGeAs**	-0.526	-0.570	**LiPbAs**	-0.025	-0.162
**NaGeAs**	0.233	0.197	**NaPbAs**	-0.014	-0.164
**KGeAs**	0.278	0.232	**KPbAs**	0.449	0.268
**RbGeAs**	0.265	0.208	**RbPbAs**	0.501	0.294
**CsGeAs**	0.313	0.238	**CsPbAs**	0.583	0.344

Table S8. The system bandgaps of the ABSb compounds (A = Li, Na, K, Rb, or Cs; B = Si, Ge, Sn, or Pb) under PBE-GGA without and with SOC. The positive system bandgap denotes a semiconducting nature while the negative one denotes a semimetallic nature.

ABX	System Bandgaps (eV)		ABX	System Bandgaps (eV)	
	without SOC	with SOC		without SOC	with SOC
**LiSiSb**	-0.347	-0.389	**LiSnSb**	-0.375	-0.503
**NaSiSb**	0.247	0.194	**NaSnSb**	-0.264	-0.403
**KSiSb**	0.528	0.461	**KSnSb**	0.259	0.143
**RbSiSb**	0.384	0.334	**RbSnSb**	0.264	0.136
**CsSiSb**	0.213	0.145	**CsSnSb**	0.333	0.172
**LiGeSb**	-0.748	-0.874	**LiPbSb**	-0.040	-0.213
**NaGeSb**	-0.019	-0.111	**NaPbSb**	-0.112	-0.369
**KGeSb**	0.210	0.119	**KPbSb**	0.313	0.056
**RbGeSb**	0.177	0.076	**RbPbSb**	0.413	0.158
**CsGeSb**	0.286	0.125	**CsPbSb**	0.480	0.197

Table S9. The system bandgaps of the ABBi compounds (A = Li, Na, K, Rb, or Cs; B = Si, Ge, Sn, or Pb) under PBE-GGA without and with SOC. The positive system bandgap denotes a semiconducting nature while the negative one denotes a semimetallic nature.

ABX	System Bandgaps (eV)		ABX	System Bandgaps (eV)	
	without SOC	with SOC		without SOC	with SOC
**LiSiBi**	-0.673	-0.810	**LiSnBi**	-0.564	-0.566
**NaSiBi**	-0.068	-0.301	**NaSnBi**	-0.582	-0.646
**KSiBi**	0.027	0.046	**KSnBi**	0.110	-0.110
**RbSiBi**	0.022	0.079	**RbSnBi**	0.154	-0.002
**CsSiBi**	0.053	0.087	**CsSnBi**	0.217	0.026
**LiGeBi**	-0.991	-0.972	**LiPbBi**	-0.526	-0.222
**NaGeBi**	-0.364	-0.496	**NaPbBi**	-0.235	-0.427
**KGeBi**	0.058	-0.008	**KPbBi**	0.102	-0.260
**RbGeBi**	0.072	0.080	**RbPbBi**	0.226	-0.199
**CsGeBi**	0.110	0.117	**CsPbBi**	0.427	-0.154

3.2. Electronic Band Structures Under PBE-GGA

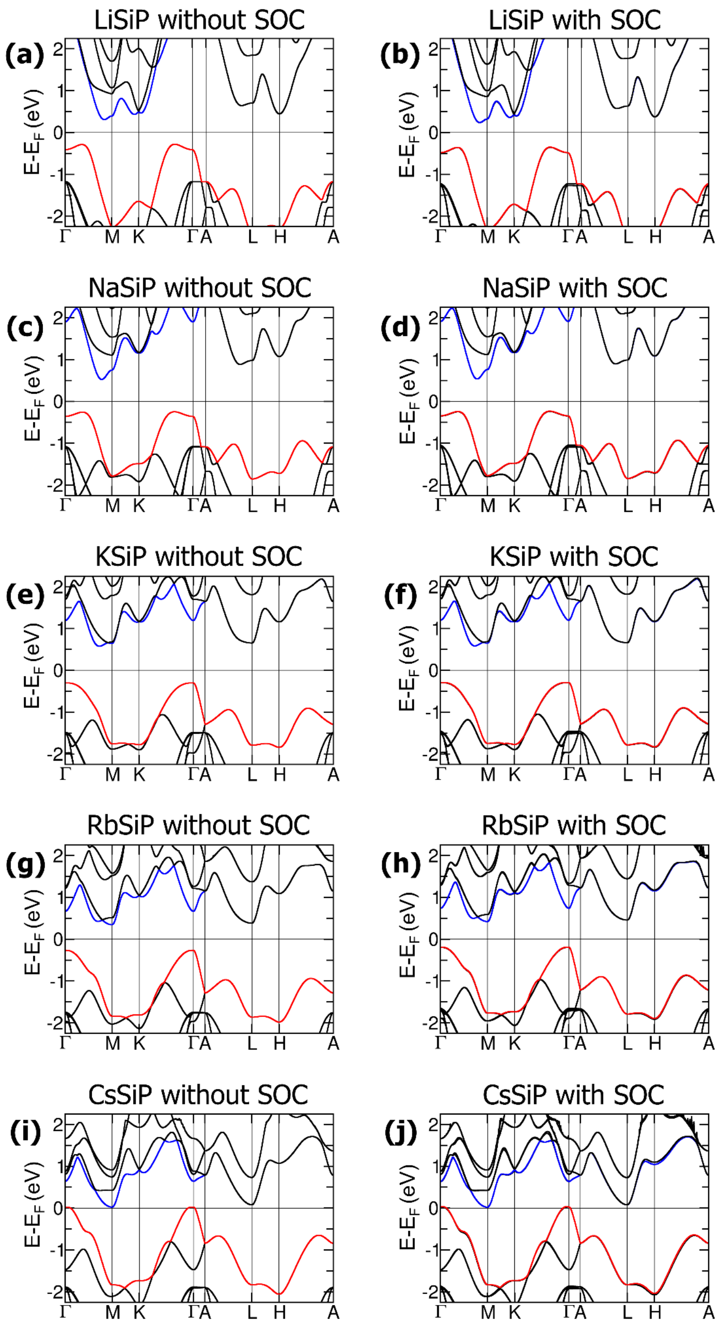

Figure S2. PBE-GGA band structures of ASiP compounds (A = Li, Na, K, Rb, or Cs) without and with the inclusion of SOC. (a, b) LiSiP, (c, d) NaSiP, (e, f) KSiP, (g, h) RbSiP, and (i, j) CsSiP.

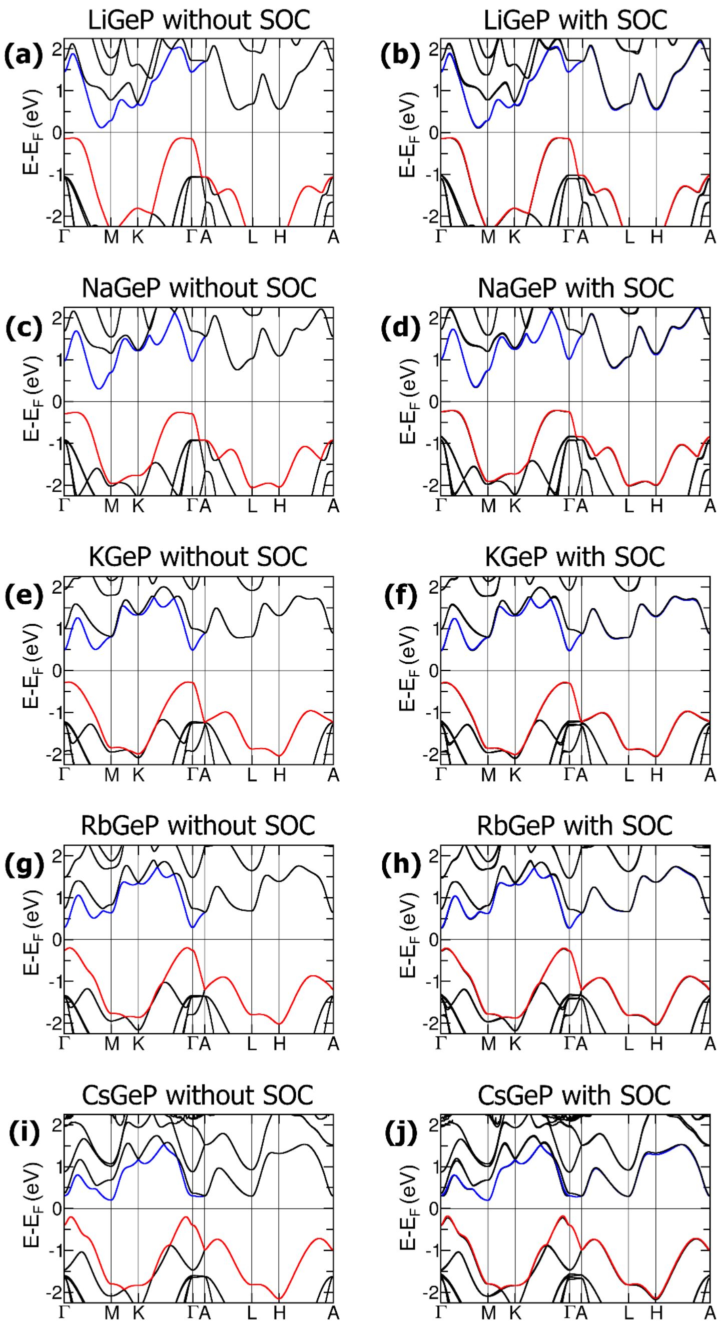

Figure S3. PBE-GGA band structures of AGeP compounds (A = Li, Na, K, Rb, or Cs) without and with the inclusion of SOC. (a, b) LiGeP, (c, d) NaGeP, (e, f) KGeP, (g, h) RbGeP, and (i, j) CsGeP.

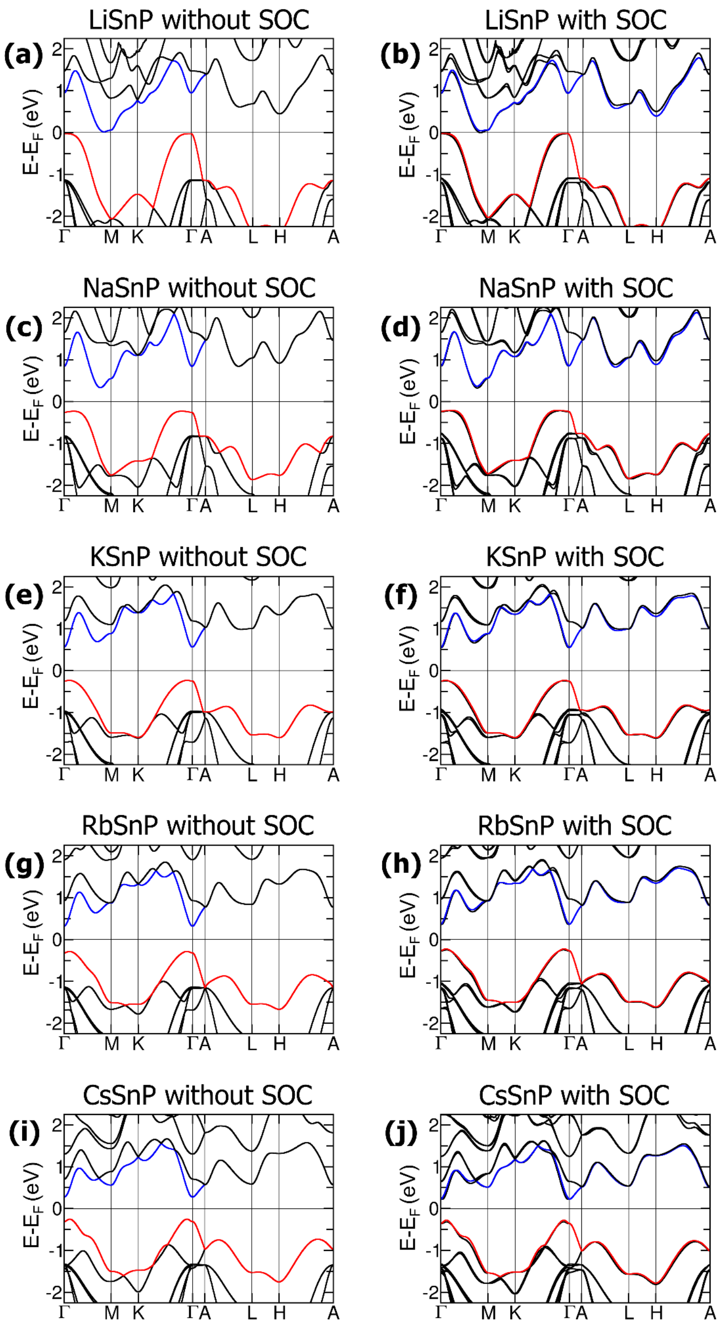

Figure S4. PBE-GGA band structures of ASnP compounds (A = Li, Na, K, Rb, or Cs) without and with the inclusion of SOC. (a, b) LiSnP, (c, d) NaSnP, (e, f) KSnP, (g, h) RbSnP, and (i, j) CsSnP.

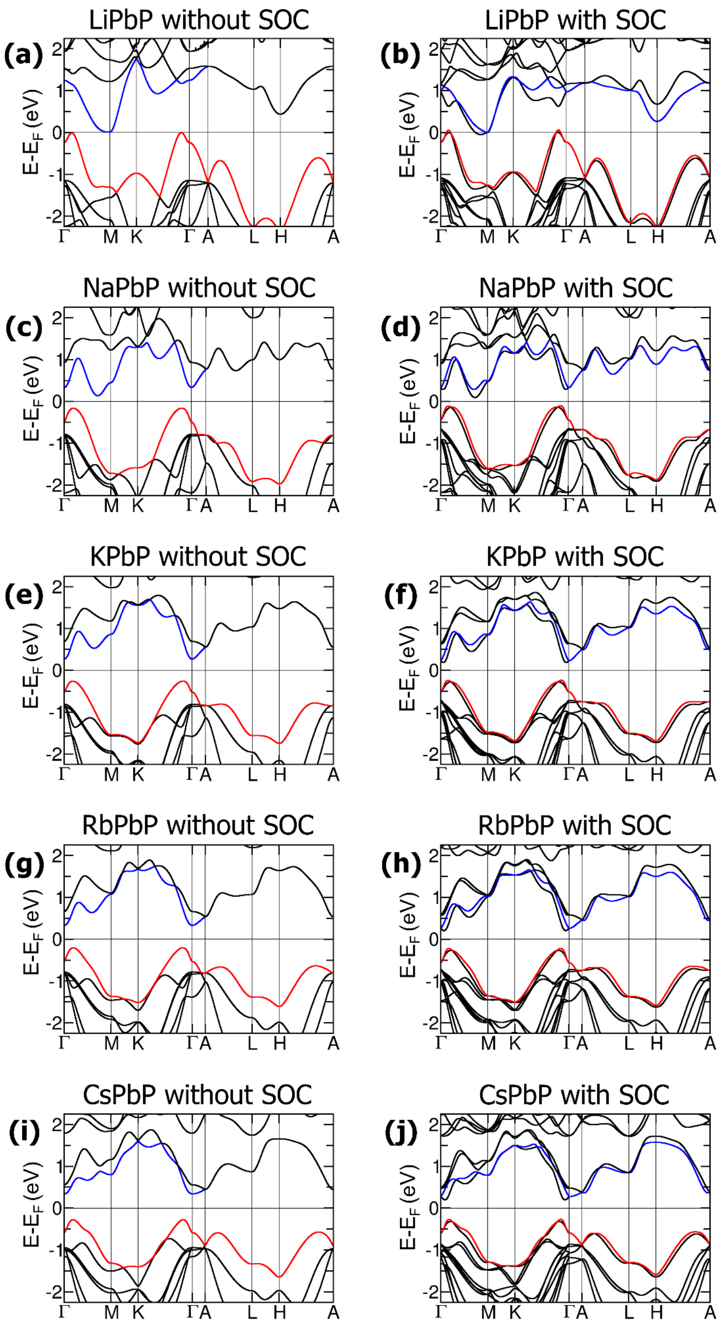

Figure S5. PBE-GGA band structures of APbP compounds (A = Li, Na, K, Rb, or Cs) without and with the inclusion of SOC. (a, b) LiPbP, (c, d) NaPbP, (e, f) KPbP, (g, h) RbPbP, and (i, j) CsPbP.

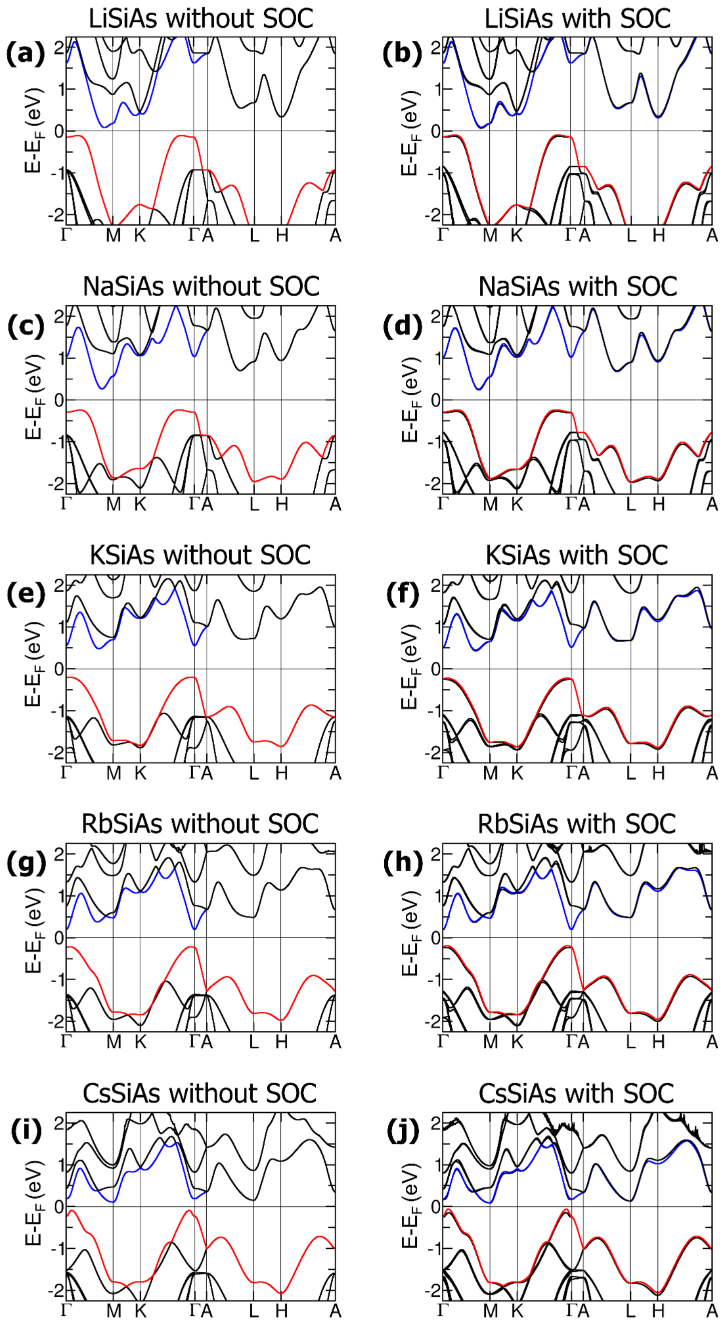

Figure S6. PBE-GGA band structures of ASiAs compounds (A = Li, Na, K, Rb, or Cs) without and with the inclusion of SOC. (a, b) LiSiAs, (c, d) NaSiAs, (e, f) KSiAs, (g, h) RbSiAs, and (i, j) CsSiAs.

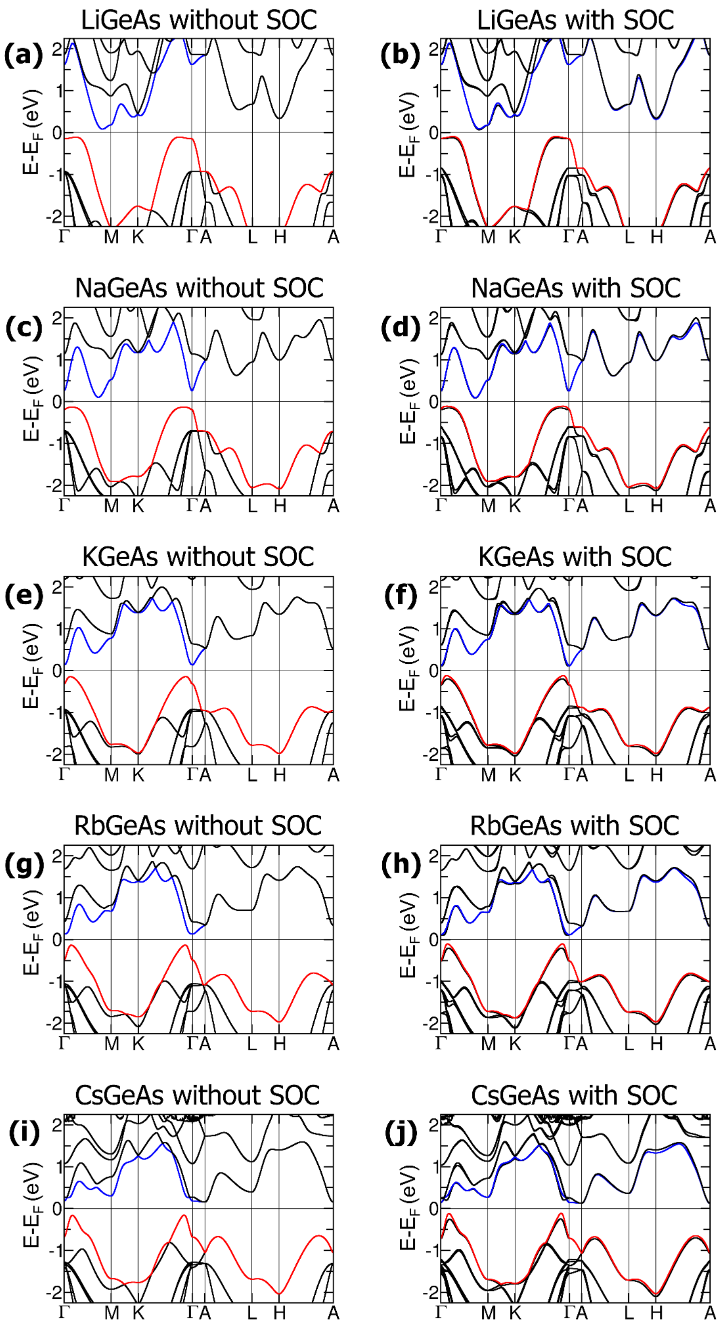

Figure S7. PBE-GGA band structures of AGeAs compounds (A = Li, Na, K, Rb, or Cs) without and with the inclusion of SOC. (a, b) LiGeAs, (c, d) NaGeAs, (e, f) KGeAs, (g, h) RbGeAs, and (i, j) CsGeAs.

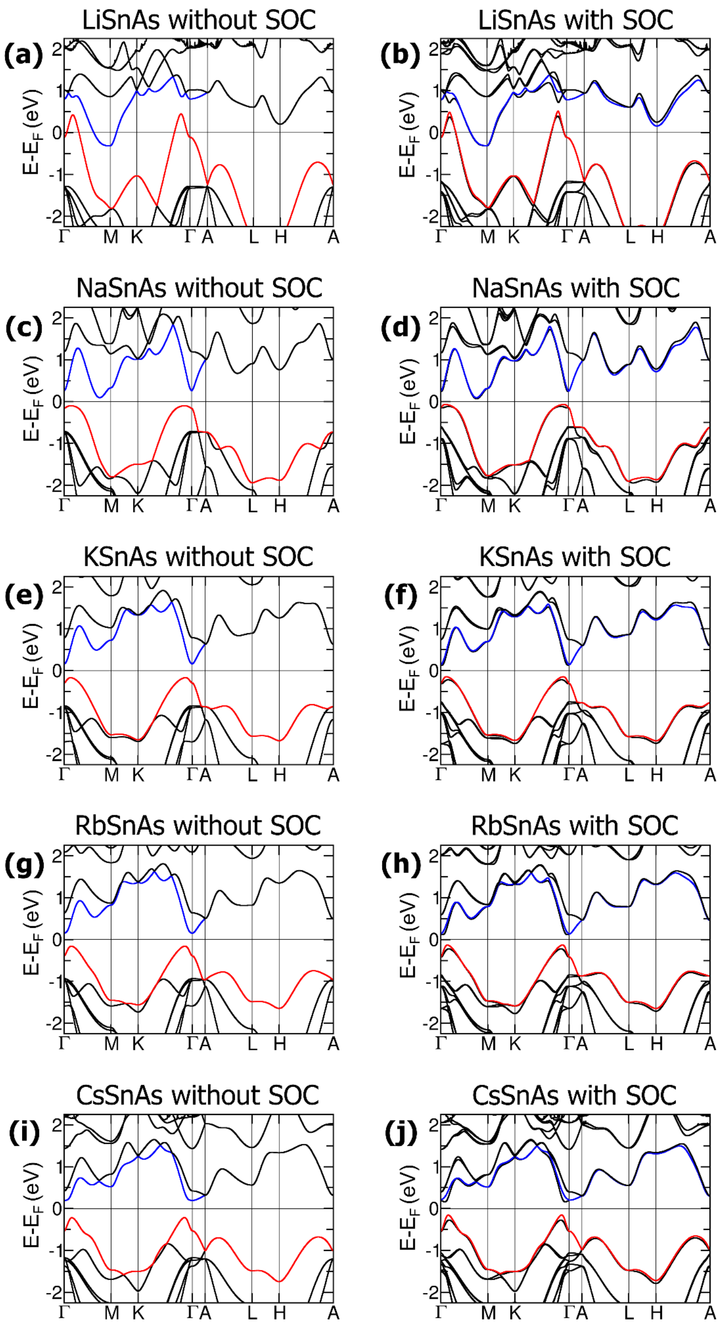

Figure S8. PBE-GGA band structures of ASnAs compounds (A = Li, Na, K, Rb, or Cs) without and with the inclusion of SOC. (a, b) LiSnAs, (c, d) NaSnAs, (e, f) KSnAs, (g, h) RbSnAs, and (i, j) CsSnAs.

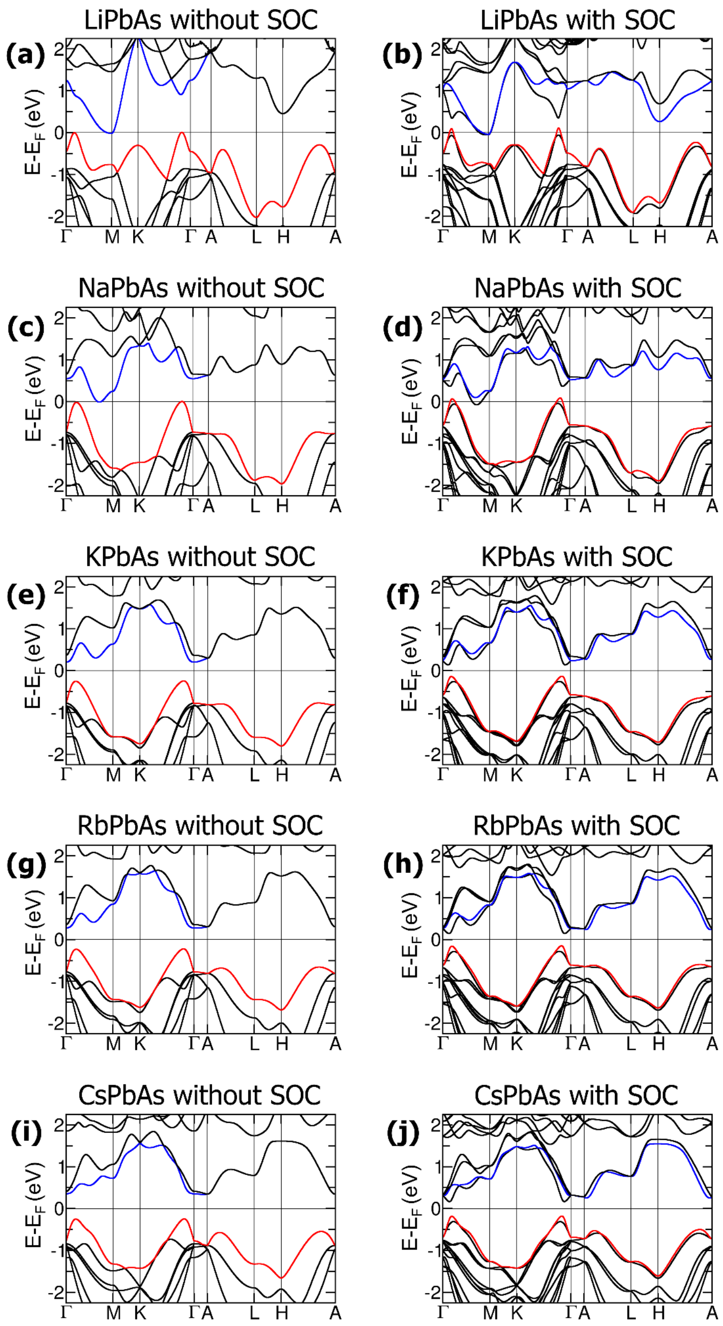

Figure S9. PBE-GGA band structures of APbAs compounds (A = Li, Na, K, Rb, or Cs) without and with the inclusion of SOC. (a, b) LiPbAs, (c, d) NaPbAs, (e, f) KPbAs, (g, h) RbPbAs, and (i, j) CsPbAs.

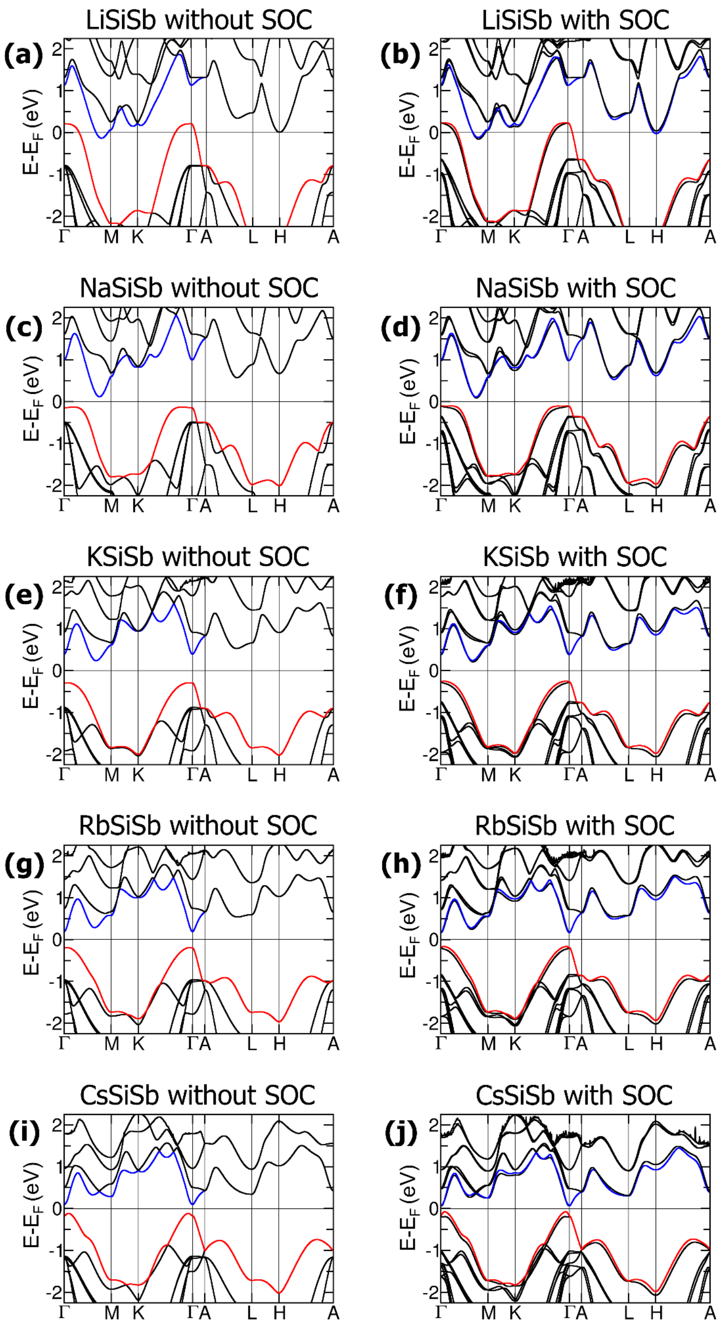

Figure S10. PBE-GGA band structures of ASiSb compounds (A = Li, Na, K, Rb, or Cs) without and with the inclusion of SOC. (a, b) LiSiSb, (c, d) NaSiSb, (e, f) KSiSb, (g, h) RbSiSb, and (i, j) CsSiSb.

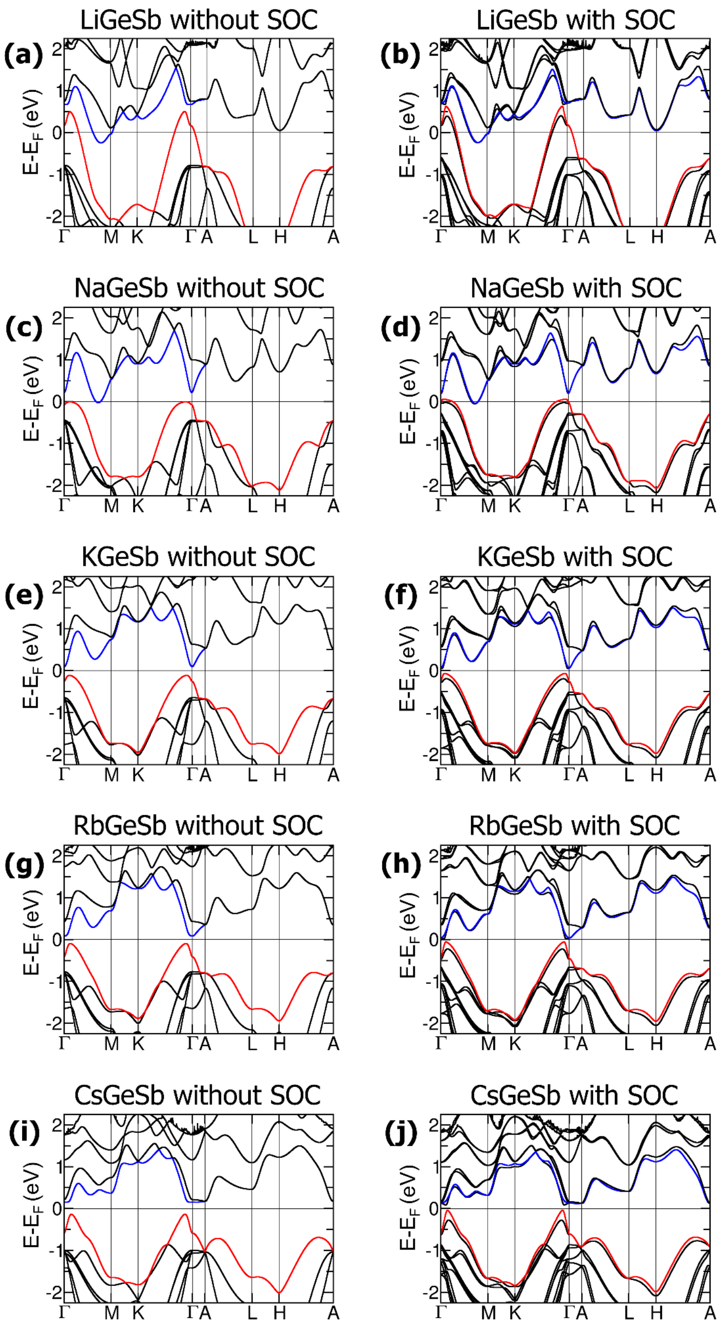

Figure S11. PBE-GGA band structures of AGeSb compounds (A = Li, Na, K, Rb, or Cs) without and with the inclusion of SOC. (a, b) LiGeSb, (c, d) NaGeSb, (e, f) KGeSb, (g, h) RbGeSb, and (i, j) CsGeSb.

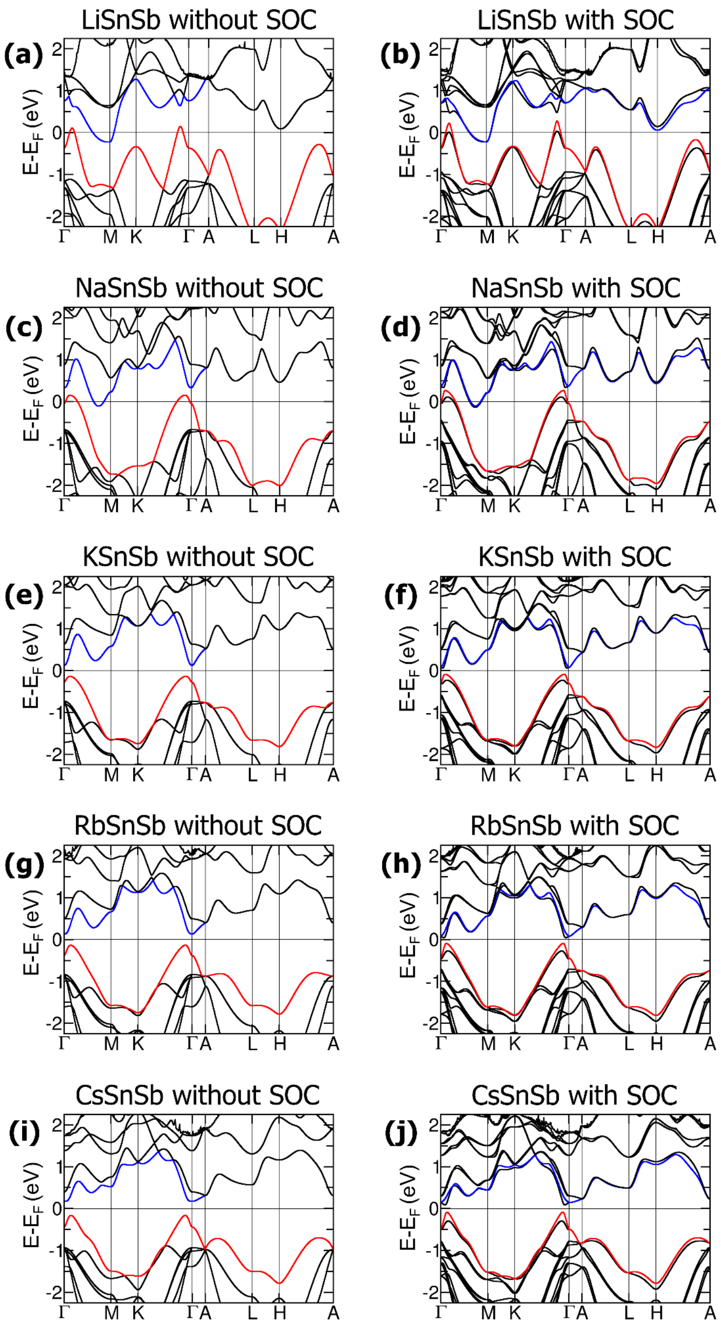

Figure S12. PBE-GGA band structures of ASnSb compounds (A = Li, Na, K, Rb, or Cs) without and with the inclusion of SOC. (a, b) LiSnSb, (c, d) NaSnSb, (e, f) KSnSb, (g, h) RbSnSb, and (i, j) CsSnSb.

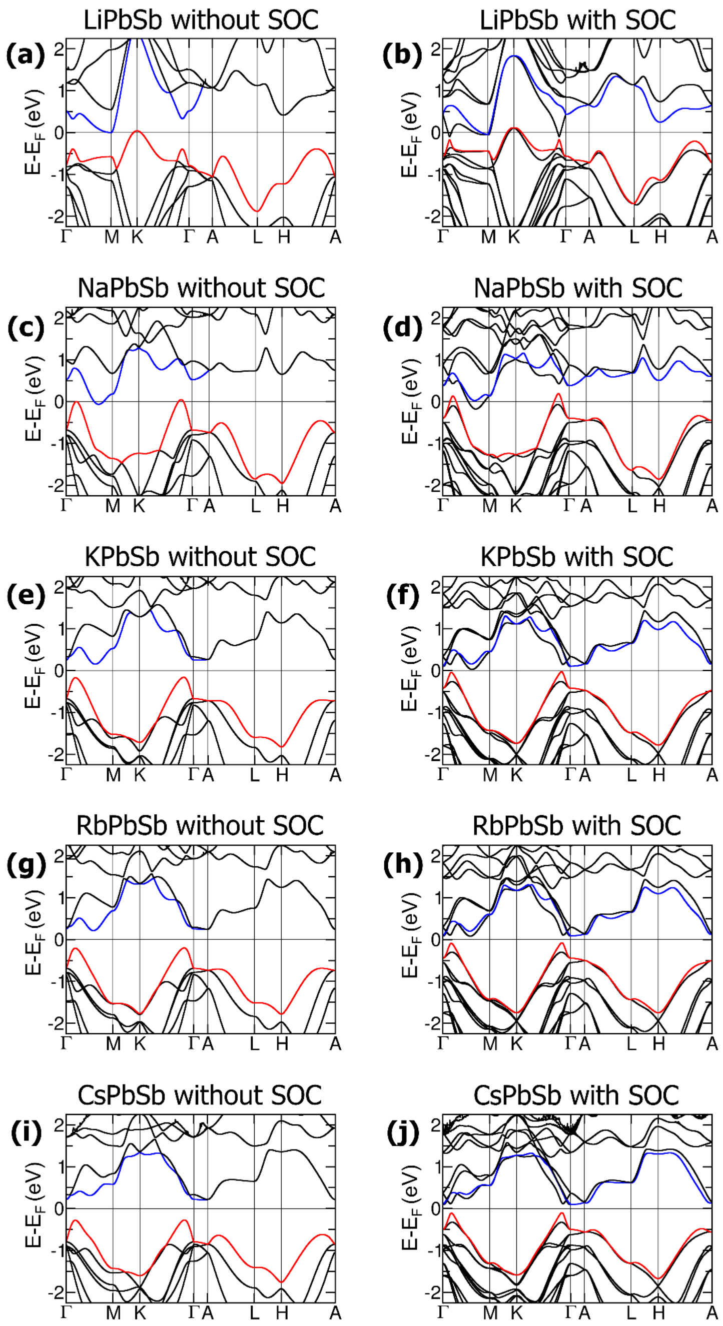

Figure S13. PBE-GGA band structures of APbSb compounds (A = Li, Na, K, Rb, or Cs) without and with the inclusion of SOC. (a, b) LiPbSb, (c, d) NaPbSb, (e, f) KPbSb, (g, h) RbPbSb, and (i, j) CsPbSb.

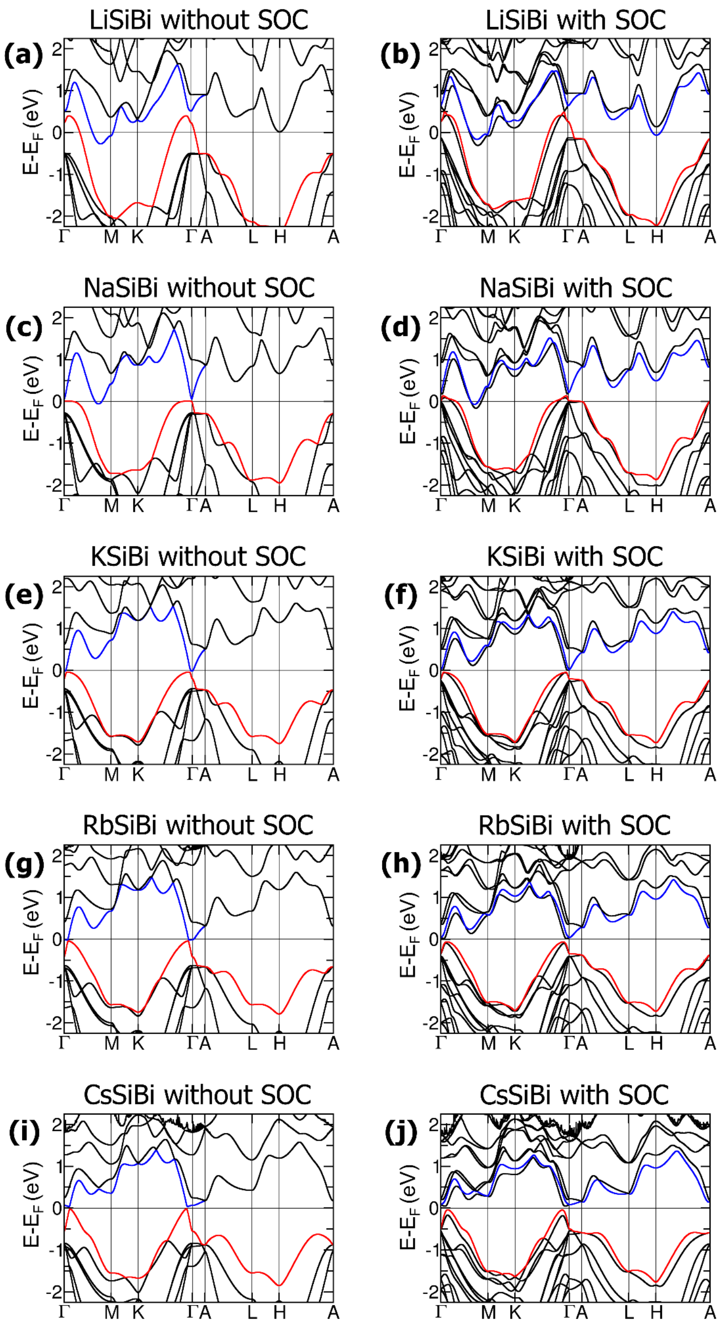

Figure S14. PBE-GGA band structures of ASiBi compounds (A = Li, Na, K, Rb, or Cs) without and with the inclusion of SOC. (a, b) LiSiBi, (c, d) NaSiBi, (e, f) KSiBi, (g, h) RbSiBi, and (i, j) CsSiBi.

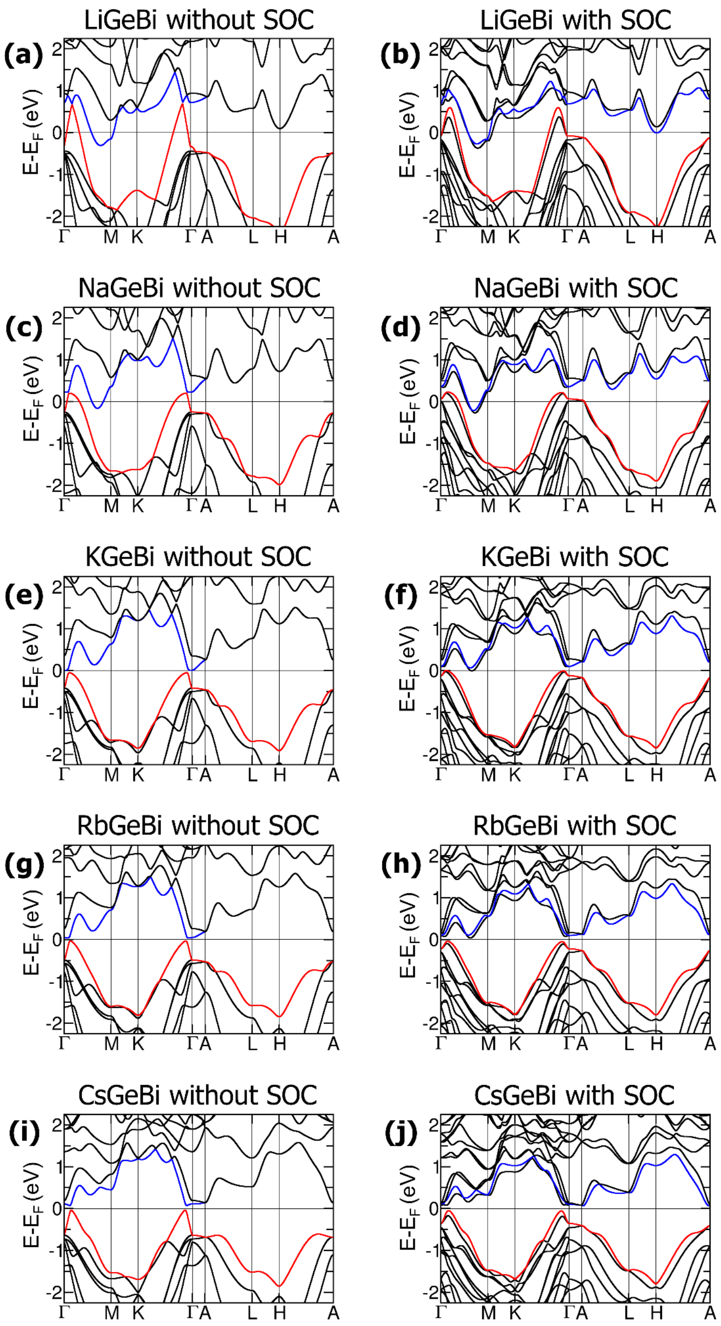

Figure S15. PBE-GGA band structures of AGeBi compounds (A = Li, Na, K, Rb, or Cs) without and with the inclusion of SOC. (a, b) LiGeBi, (c, d) NaGeBi, (e, f) KGeBi, (g, h) RbGeBi, and (i, j) CsGeBi.

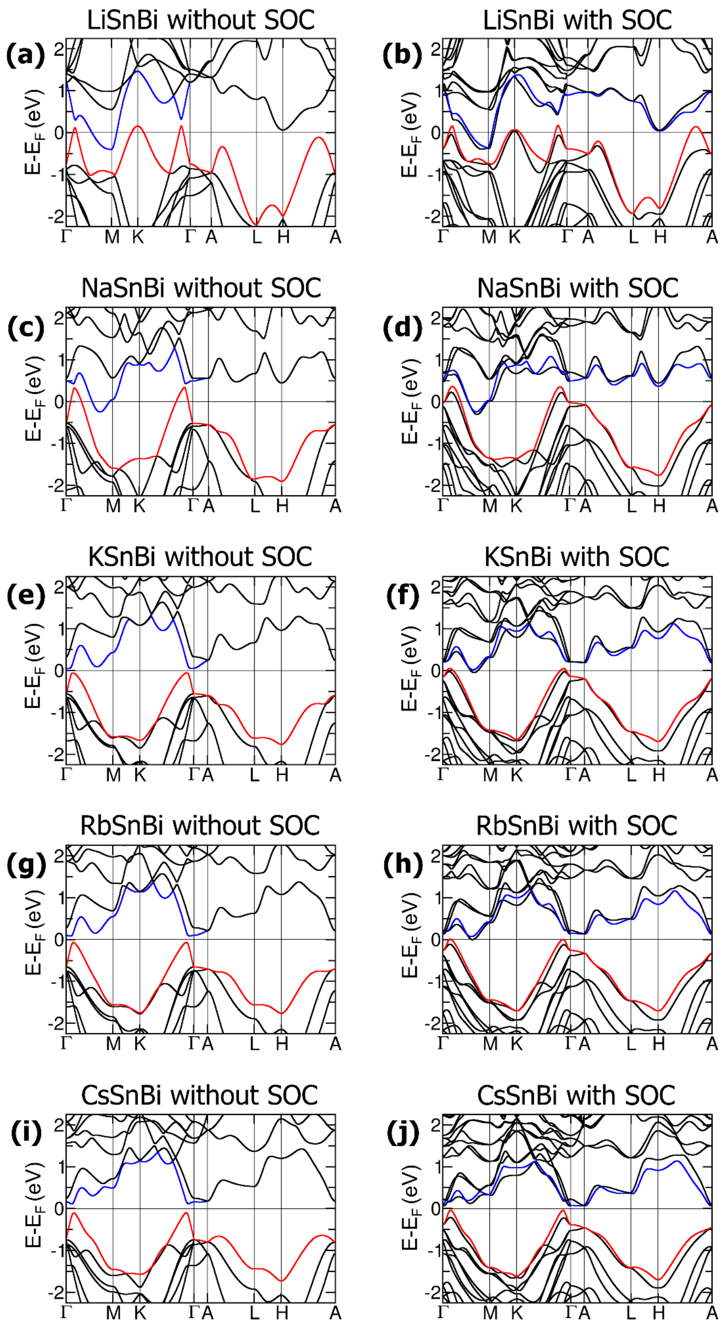

Figure S16. PBE-GGA band structures of ASnBi compounds (A = Li, Na, K, Rb, or Cs) without and with the inclusion of SOC. (a, b) LiSnBi, (c, d) NaSnBi, (e, f) KSnBi, (g, h) RbSnBi, and (i, j) CsSnBi.

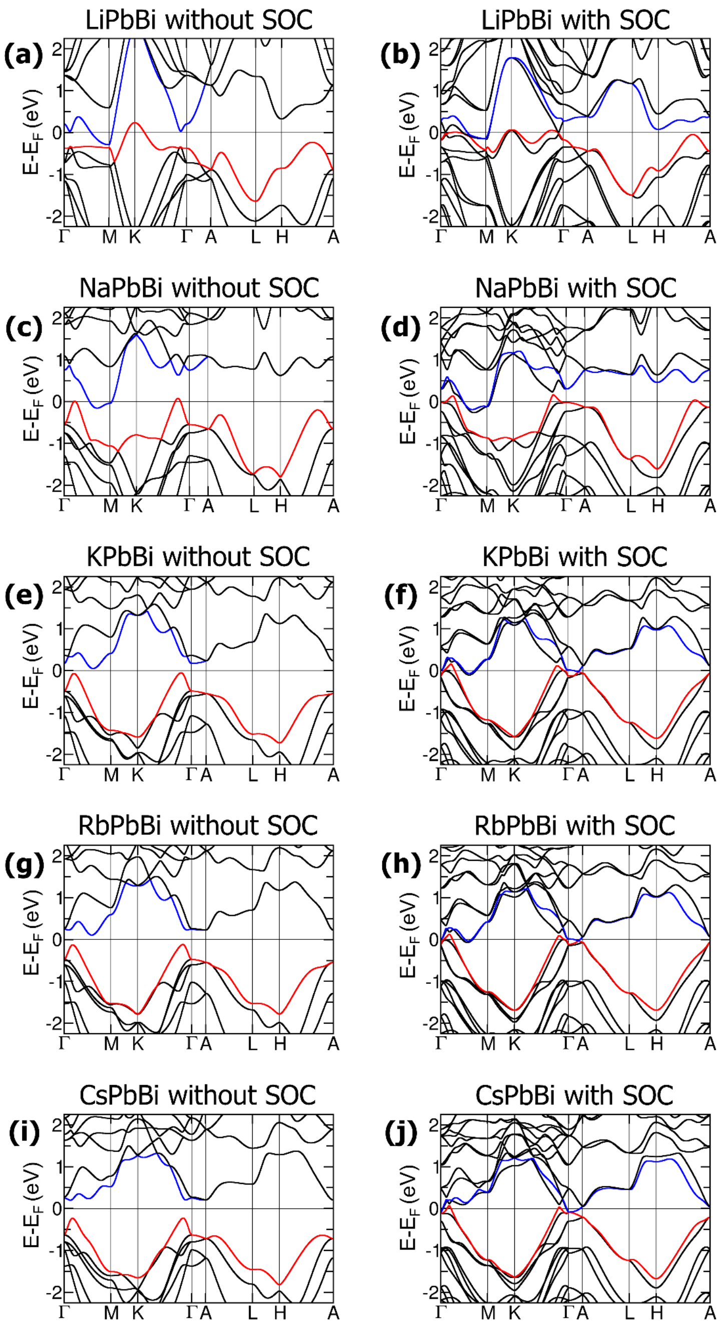

Figure S17. PBE-GGA band structures of APbBi compounds (A = Li, Na, K, Rb, or Cs) without and with the inclusion of SOC. (a, b) LiPbBi, (c, d) NaPbBi, (e, f) KPbBi, (g, h) RbPbBi, and (i, j) CsPbBi.

3.3. Electronic Band Structures Under HSE06

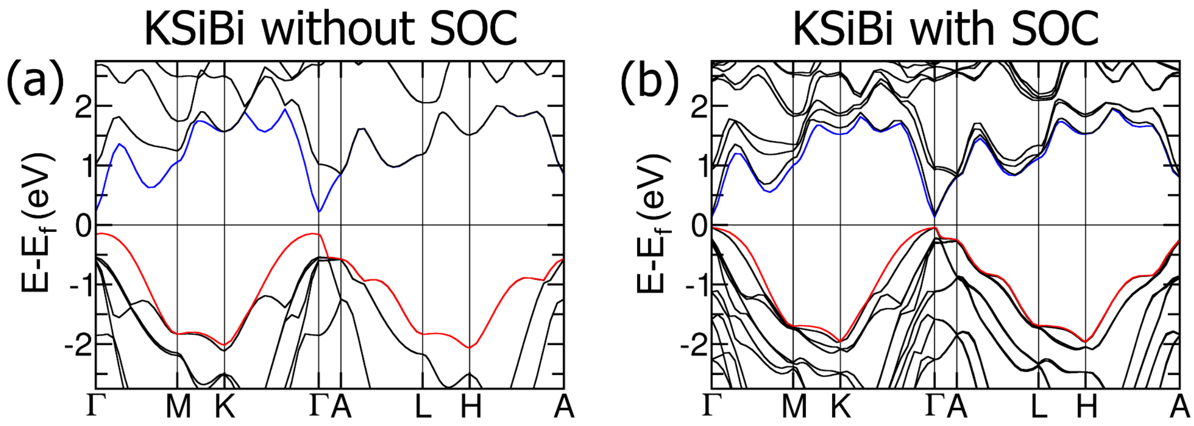

Figure S18. HSE06 band structures of KSiBi (a) without and (b) with SOC.

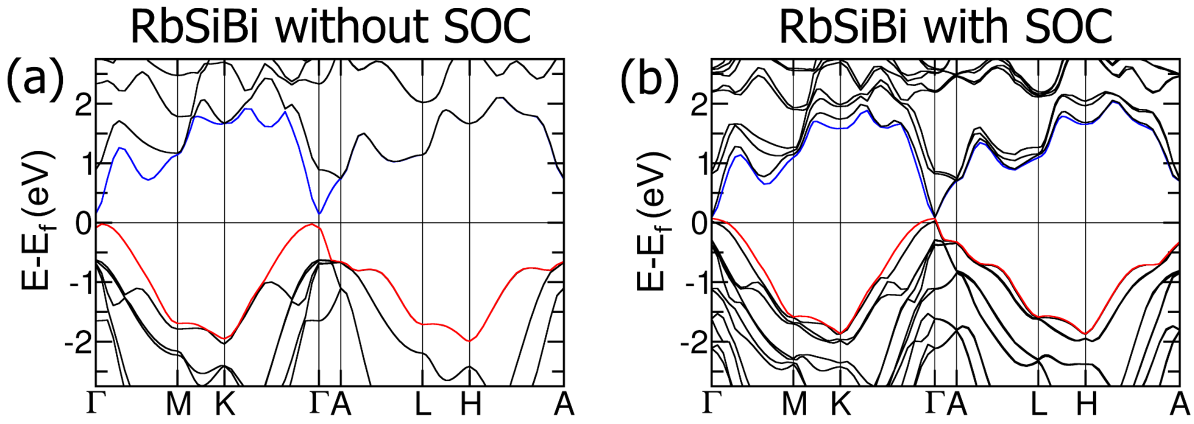

Figure S19. HSE06 band structures of RbSiBi (a) without and (b) with SOC.

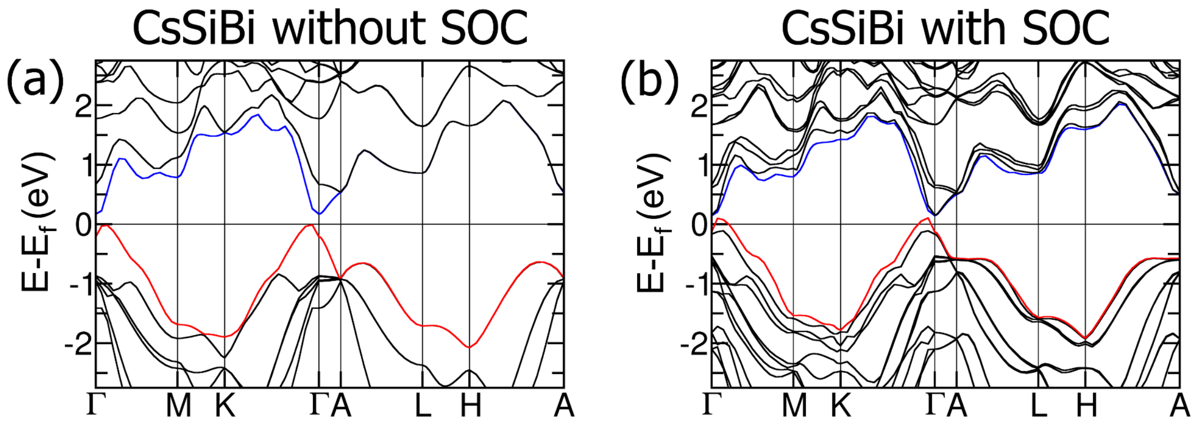

Figure S20. HSE06 band structures of CsSiBi (a) without and (b) with SOC.

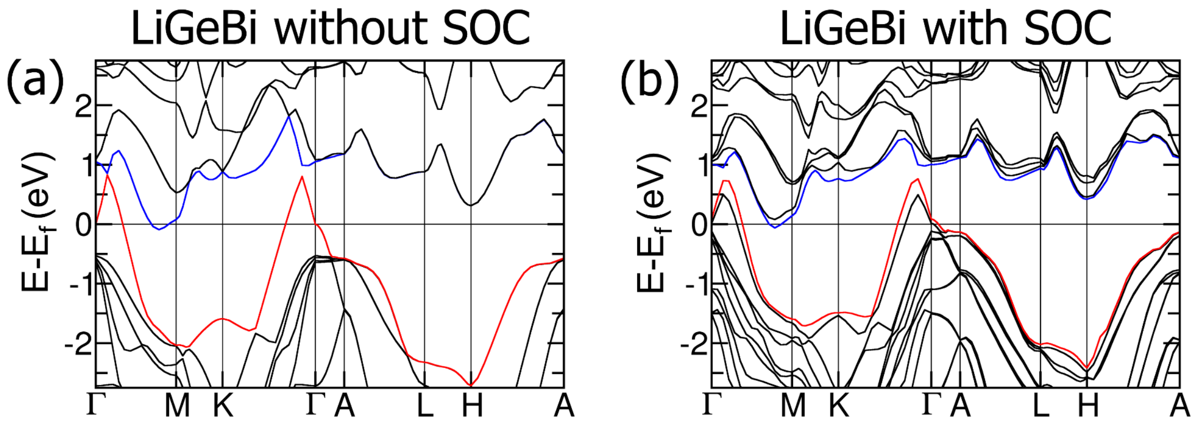

Figure S21. HSE06 band structures of LiGeBi (a) without and (b) with SOC.

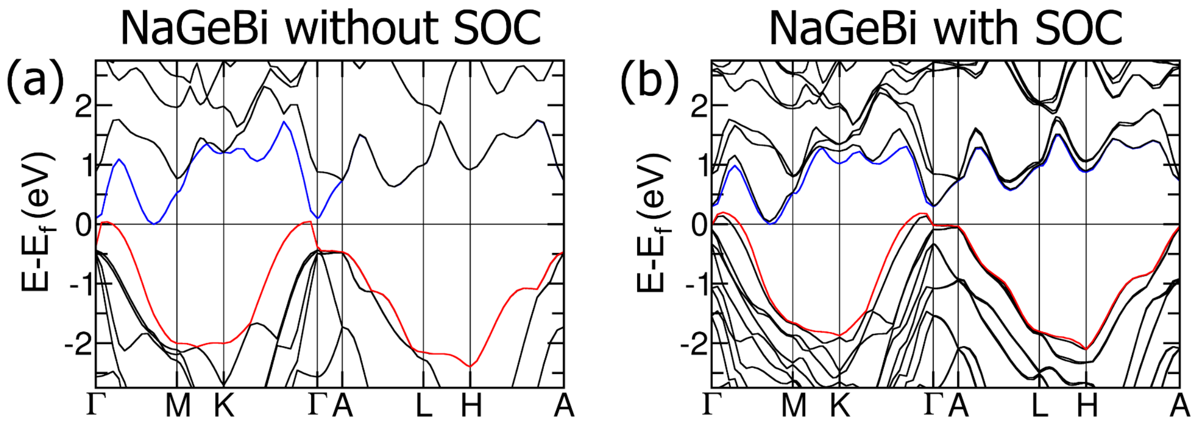

Figure S22. HSE06 band structures of NaGeBi (a) without and (b) with SOC.

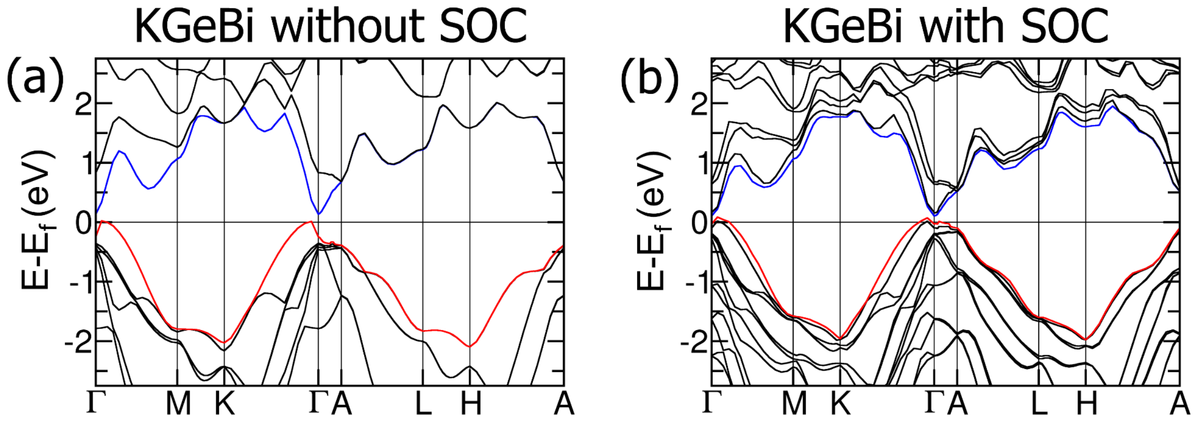

Figure S23. HSE06 band structures of KGeBi (a) without and (b) with SOC.

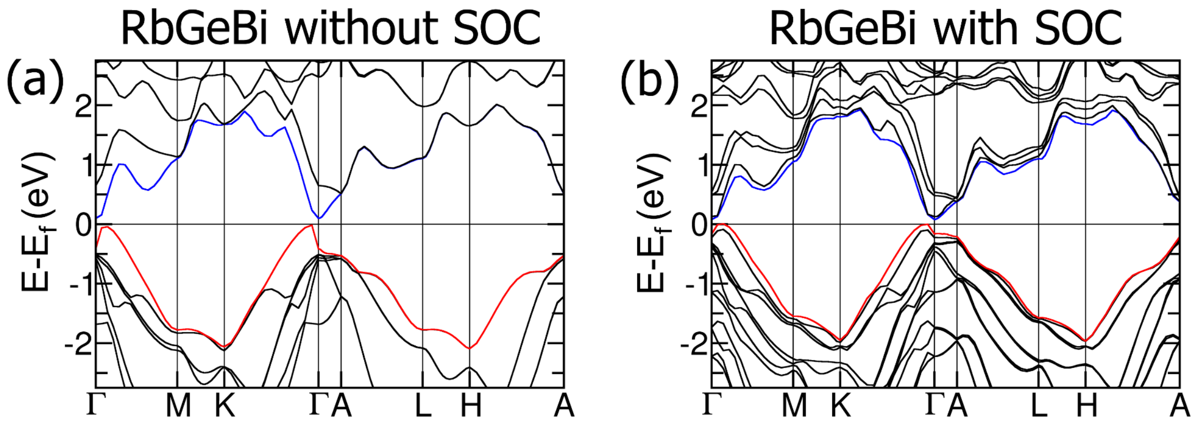

Figure S24. HSE06 band structures of RbGeBi (a) without and (b) with SOC.

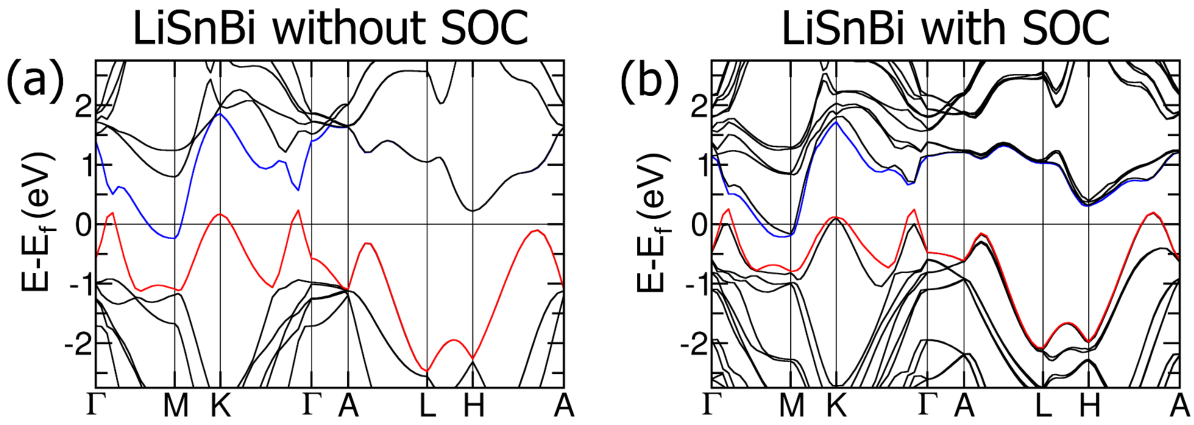

Figure S25. HSE06 band structures of LiSnBi (a) without and (b) with SOC.

3.4. Effect of Adjusting the Spin-Orbit Coupling Strength (SOC)

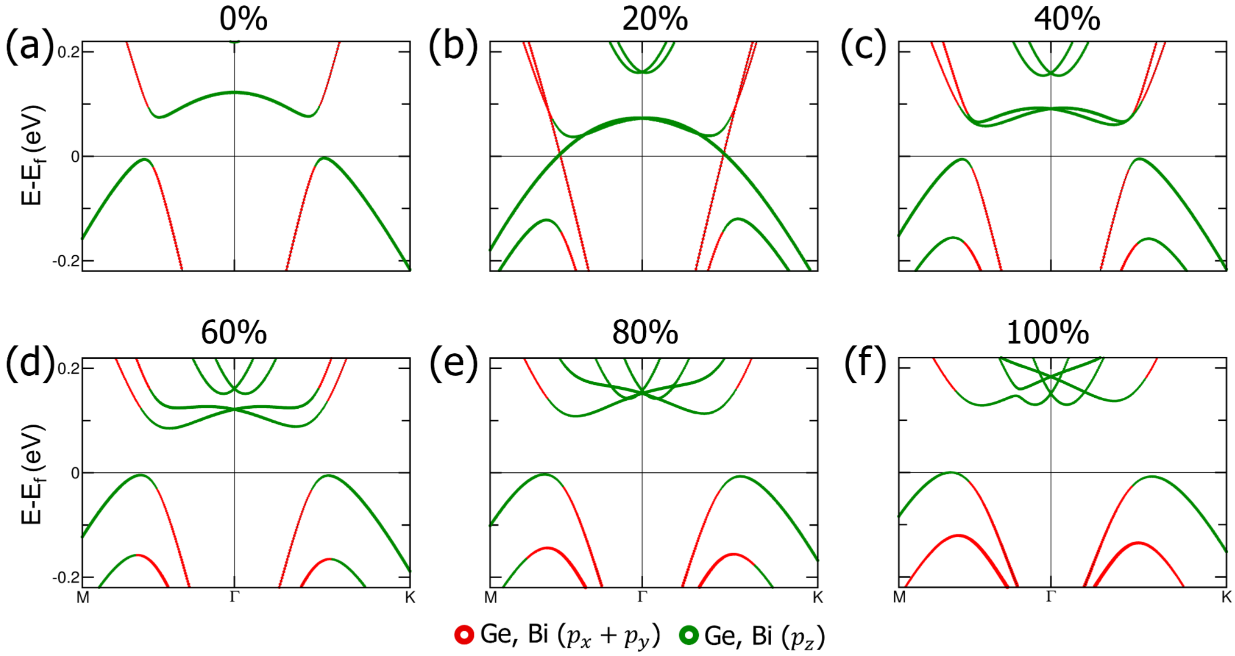

Figure S26. Effect of adjusting the strength of SOC on the bulk electronic states with orbital projection for CsGeBi. Band structures of CsGeBi with (a) 0%, (b) 20%, (c) 40%, (d) 60%, (e) 80%, and (f)100% of the real atomic SOC.

3.5. Comparison of HSE06 and Wannier Band Structures

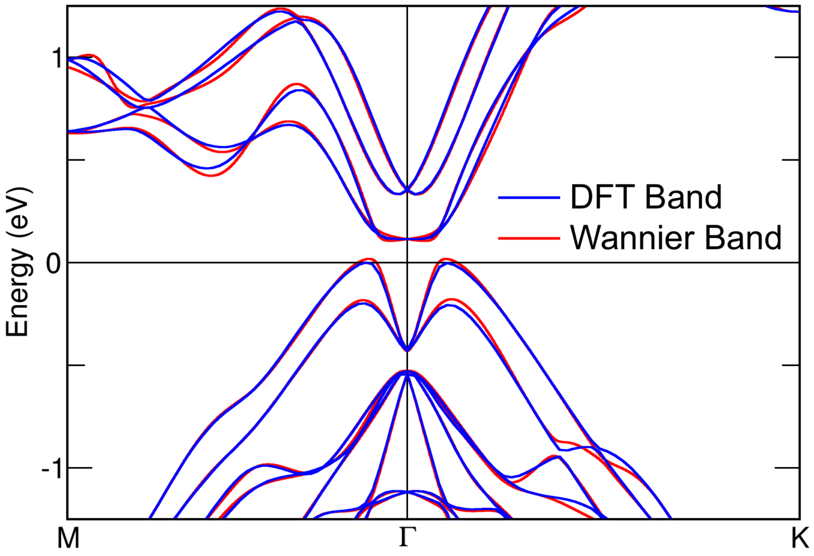

Figure S27. Comparison of the HSE06 and Wannier band structures of CsGeBi with SOC.

In our calculations of the topological properties based on Wannier functions, we carefully selected the projection orbitals to ensure accurate fitting. We utilized the *s* and *p* (p_x, p_y, p_z) orbitals of the Cs, Ge, and Bi atoms. With the use of these orbitals, we were able to obtain a good fitting of Wannier functions consistent with the DFT electronic properties, as shown in **Figure S27**.

3.6. Surface Electronic Spectrum

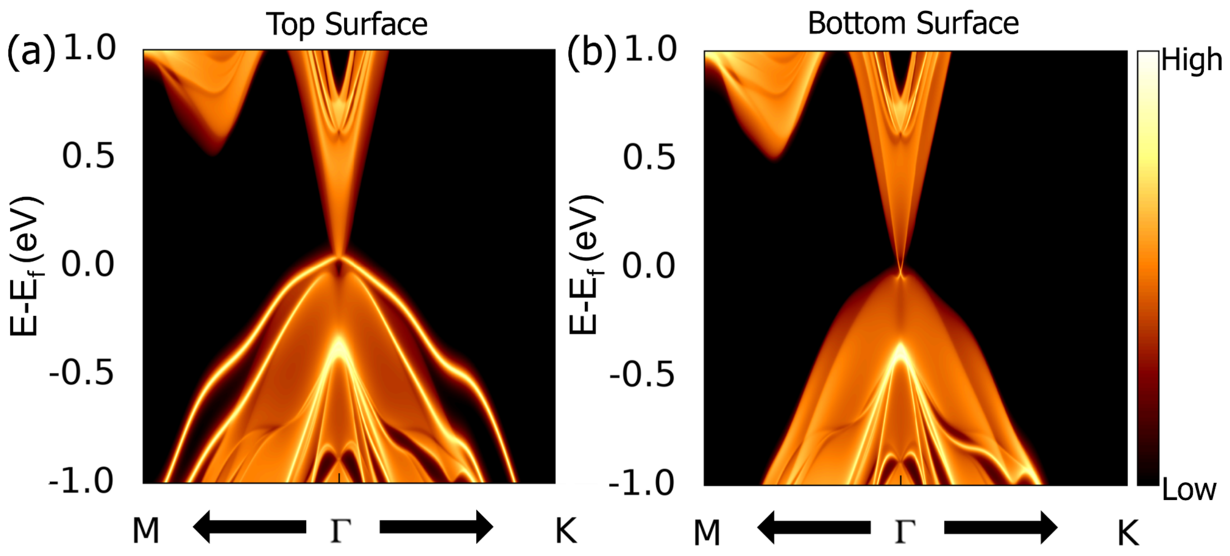

Figure S28. The surface electronic spectrum of RbSiBi under HSE06 at (a) the top surface and (b) the bottom surface along the (001) surface.

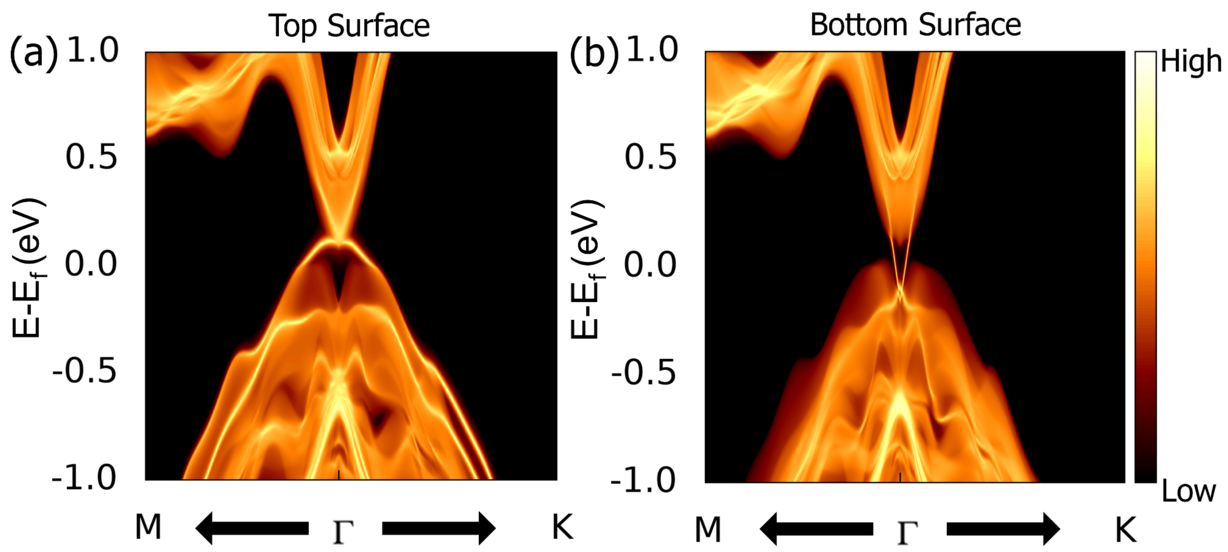

Figure S29. The surface electronic spectrum of CsSiBi under HSE06 at (a) the top surface and (b) the bottom surface along the (001) surface.

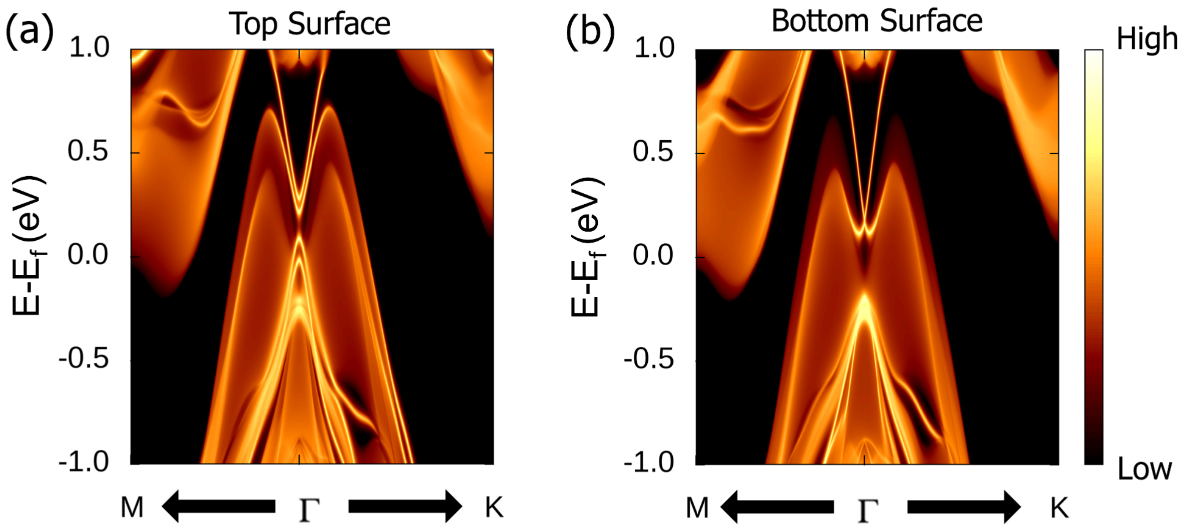

Figure S30. The surface electronic spectrum of LiGeBi under HSE06 at (a) the top surface and (b) the bottom surface along the (001) surface.

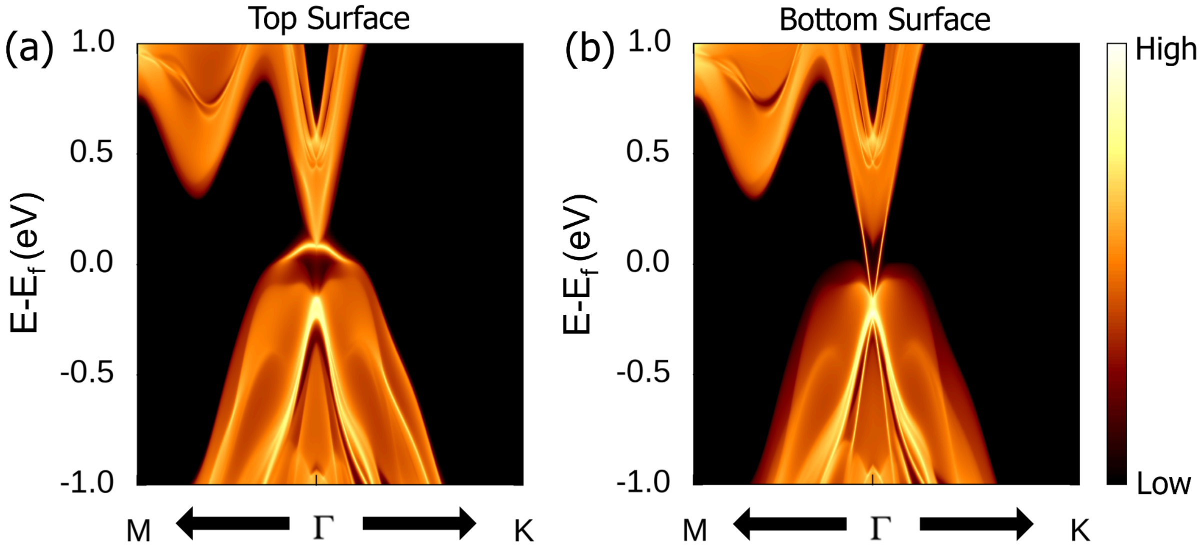

Figure S31. The surface electronic spectrum of KGeBi under HSE06 at (a) the top surface and (b) the bottom surface along the (001) surface.

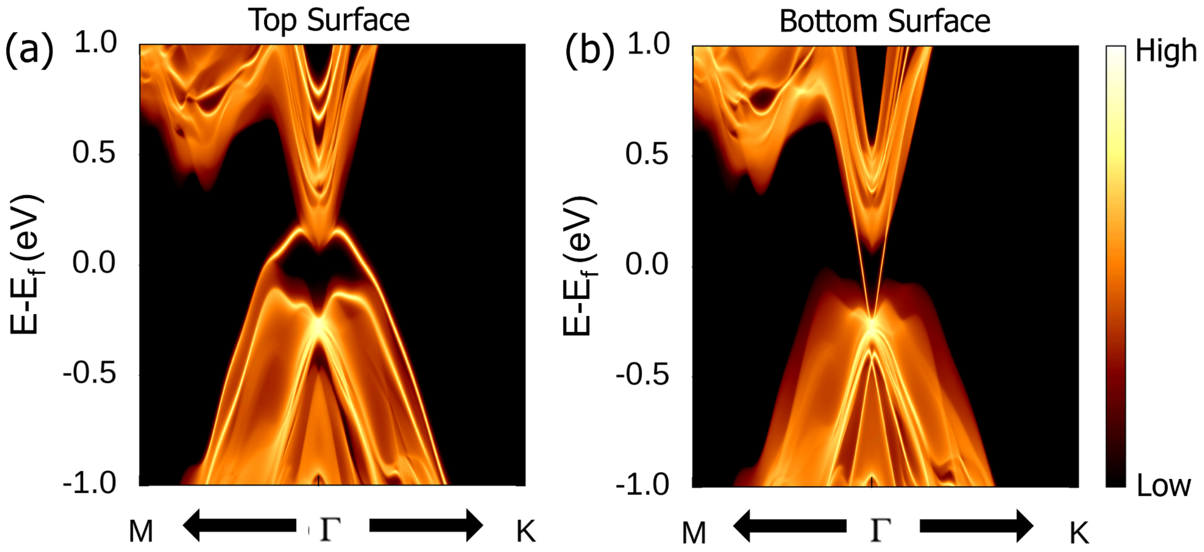

Figure S32. The surface electronic spectrum of RbGeBi under HSE06 at (a) the top surface and (b) the bottom surface along the (001) surface.

4. Structural Stability of Topological ABX Zintl Compounds

4.1. Phonon Dispersion Spectra of Topologically Nontrivial ABX Zintl Compounds

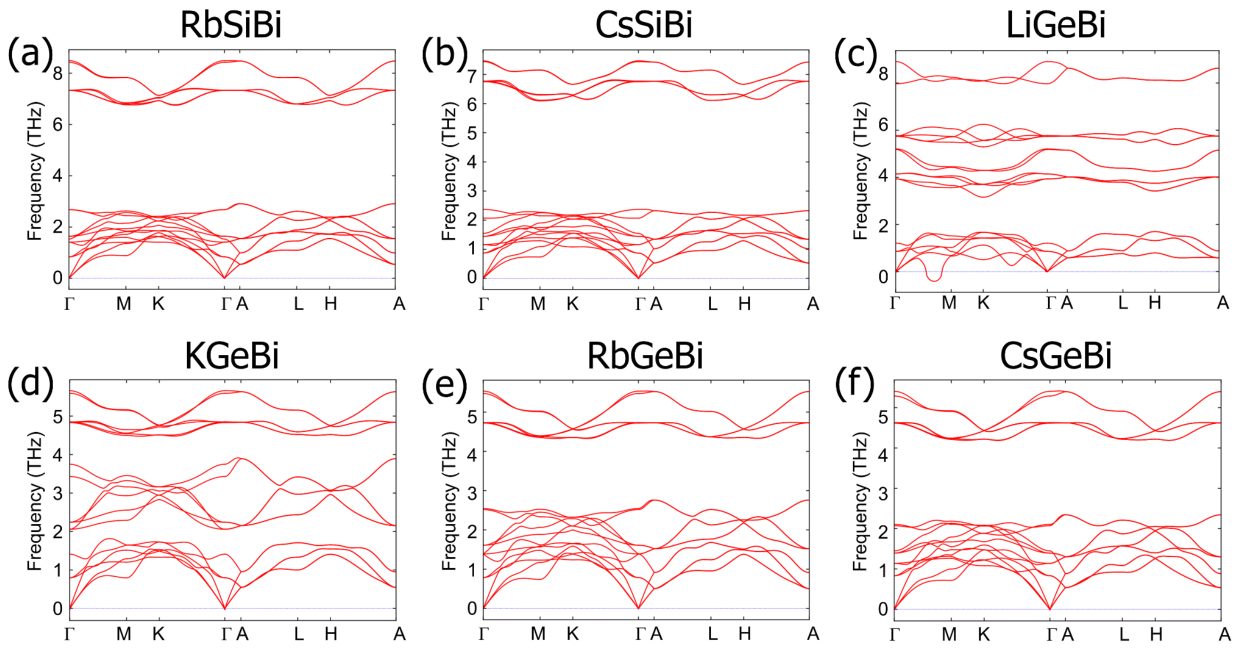

Figure S33. Phonon dispersions of the topologically nontrivial ABX compounds: (a) RbSiBi, (b) CsSiBi, (c) LiGeBi, (d) KGeBi, (e) RbGeBi, and (f) CsGeBi.

4.2. Formation Energies of Topologically Nontrivial ABX Zintl Compounds

To further support the structural stability of the six topologically nontrivial ABX Zintl compounds, their formation energies were calculated via the formula:

$$E_{form}(ABX) = E_{ABX} - (E_A + E_B + E_X) \qquad \text{(Eq. 1)}$$

where E_{ABX} is the total energy of the ABX compound, E_A, E_B, and E_X are the chemical potentials of the A = Rb, Cs, Li, or K; B = Si or Ge; and X = Bi atoms, respectively, in the bulk phase.

Table S10. The formation energies of the topologically nontrivial ABX Zintl compounds.

ABX	Formation Energy (eV)
**RbSiBi**	-0.762
**CsSiBi**	-0.052
**LiGeBi**	-0.370
**KGeBi**	-0.604
**RbGeBi**	-1.261
**CsGeBi**	-0.576

4.3. Mechanical Stability of CsGeBi

Table S11. The elastic constants and elastic moduli of the representative CsGeBi compound.

**Elastic Stiffness Constants (C_{ij})**	
C_{11}	39.209
C_{12}	16.808
C_{13}	12.215
C_{33}	38.868
C_{44}	13.055
C_{66}	11.201
**Elastic Modulus**	
Bulk modulus (B, GPa)	22.164
Shear modulus (G, GPa)	12.475
Young's modulus (E, GPa)	31.512
Pugh's ratio (B/G)	1.777
Poisson's ratio (ν)	0.263

The bulk modulus (B), shear modulus (G), Young's modulus (E), and Pugh's ratio (B/G) were calculated for CsGeBi and the results of the elastic modulus calculation are summarized in **Table S11**. The ratio of bulk modulus (B) and shear modulus (G) can be used to evaluate the brittleness and ductility of crystals. When Pugh's ratio (B/G) is less than 1.75, then, the material is brittle conversely, when it is greater than 1.75, then it is considered ductile. The Pugh's ratio of CsGeBi is 1.78 which denotes it as a ductile compound.
